# An ensemble of mathematical models showing diauxic growth behaviour

**DOI:** 10.1186/s12918-018-0604-8

**Published:** 2018-09-21

**Authors:** Andreas Kremling, Johannes Geiselmann, Delphine Ropers, Hidde de Jong

**Affiliations:** 1 Systems Biotechnology, Technical University of Munich, Boltzmannstrasse 15, Garching b. München, 85748 Germany; 2 Laboratoire Interdisciplinaire de Physique, Université Grenoble Alpes, 140 avenue de la Physique, Saint Martin d’Hères, 38402 France; 3 Inria, Université Grenoble Alpes, 655 avenue de l’Europe, Saint Ismier Cedex, 38334 France

**Keywords:** Carbon catabolite repression, Dynamical modeling, Flux balance models, Kinetic models, Resource allocation models, *Escherichia coli*

## Abstract

**Background:**

Carbon catabolite repression (CCR) controls the order in which different carbon sources are metabolised. Although this system is one of the paradigms of regulation in bacteria, the underlying mechanisms remain controversial. CCR involves the coordination of different subsystems of the cell - responsible for the uptake of carbon sources, their breakdown for the production of energy and precursors, and the conversion of the latter to biomass. The complexity of this integrated system, with regulatory mechanisms cutting across metabolism, gene expression, and signalling, has motivated important modelling efforts over the past four decades, especially in the enterobacterium *Escherichia coli*.

**Results:**

Starting from a simple core model with only four intracellular metabolites, we develop an ensemble of model variants, all showing diauxic growth behaviour during a batch process. The model variants fall into one of the four categories: flux balance models, kinetic models with growth dilution, kinetic models with regulation, and resource allocation models. The model variants differ from one another in only a single aspect, each breaking the symmetry between the two substrate assimilation pathways in a different manner, and can be quantitatively compared using a so-called diauxic growth index. For each of the model variants, we predict the behaviour in two new experimental conditions, namely a glucose pulse for a culture growing in minimal medium with lactose and a batch culture with different initial concentrations of the components of the transport systems. When qualitatively comparing these predictions with experimental data for these two conditions, a number of models can be excluded while other model variants are still not discriminable. The best-performing model variants are based on inducer inclusion and activation of enzymatic genes by a global transcription factor, but the other proposed factors may complement these well-known regulatory mechanisms.

**Conclusions:**

The model ensemble presented here offers a better understanding of the variety of mechanisms that have been proposed to play a role in CCR. In addition, it provides an educational resource for systems biology, as it gives an introduction to a broad range of modeling approaches in the context of a simple but biologically relevant example.

**Electronic supplementary material:**

The online version of this article (10.1186/s12918-018-0604-8) contains supplementary material, which is available to authorized users.

## Background

**Fig. 1:**
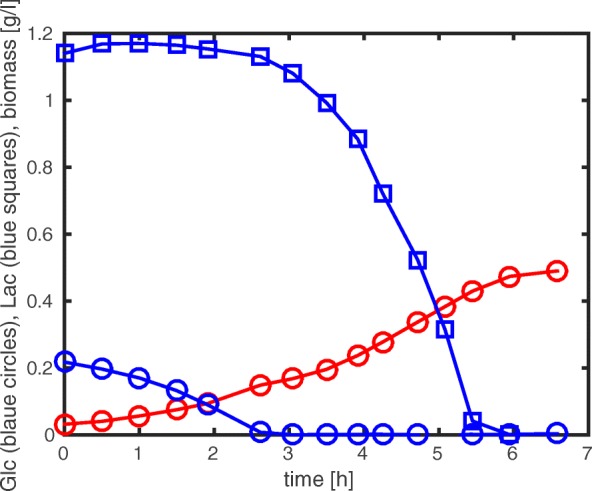
Diauxic growth as a typical manifestation of carbon catabolite repression in bacteria (experimental data are taken from [14]). The plot shows the sequential uptake of glucose (blue circles) and lactose (blue squares) by growing *Escherichia coli* bacteria on a mixture of carbon sources. This leads to the two-stage accumulation of biomass (red circles) at a high growth rate (on glucose) and a lower growth rate (on lactose) until all carbon sources have been exhausted

Carbon catabolite repression (CCR) is the main mechanism controlling carbohydrate uptake in bacteria, and therefore also controlling whether or not different carbon sources are metabolized in parallel or sequentially. Although described as a paradigm of the regulation of bacterial metabolism, the underlying mechanisms remain controversial (see [1, 2]). The system shows a high level of complexity comprising metabolic, gene expression, and signal processing. A typical example of CCR is the phenomenon of diauxic growth (Fig. 1).

Different hypotheses concerning the dynamic functioning of the system have been explored by a variety of modelling approaches [2]. The aim of this study is to compare these hypotheses and their ability to capture some key characteristics of CCR within a single modeling framework. To this end, based on a (simple) core model structure with only four intracellular metabolites, we developed an ensemble of model variants, all showing diauxic growth behaviour during batch cultivation with two substrates. The model variants differ in only a few structural properties and have only a small number of free parameters. The use of small models with few parameters allows us to focus on the underlying network structure when comparing the different model variants.

**Fig. 2:**
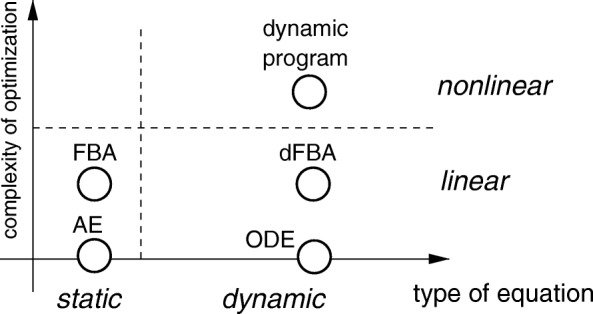
Overview of the ensemble modeling strategy employed in this study. We do not only distinguish between the type of equations of the model (static or dynamic), but also take into account mechanistic models *vs* models based on (linear or nonlinear) optimization. The vertical axis reflects the increasing complexity of the optimization program: a non-linear problem is more difficult to solve than a linear program. The zero of this axis corresponds to models without optimization. Abbreviations used: AE (algebraic equations), ODE (ordinary differential equations), FBA (flux balance analysis), dFBA (dynamic flux balance analysis)

Ensemble modeling approaches have been used to explore and analyze different model structures and/or different set of parameters (see [3, 4] for examples of ensemble modelling). As can be seen in Fig. 2, we extend the scope of ensemble modelling in the present study by adding another dimension. Instead of restricting the ensemble of model variants to static or dynamic models that quantitatively describe the mechanisms of carbohydrate assimilation and its regulation, we also introduce model variants that make up for a lack of mechanistic information by using different (linear and nonlinear) optimization programs, applied either statistically or dynamically. A major representative of such optimization-based models are flux balance models [5, 6].

**Fig. 3:**
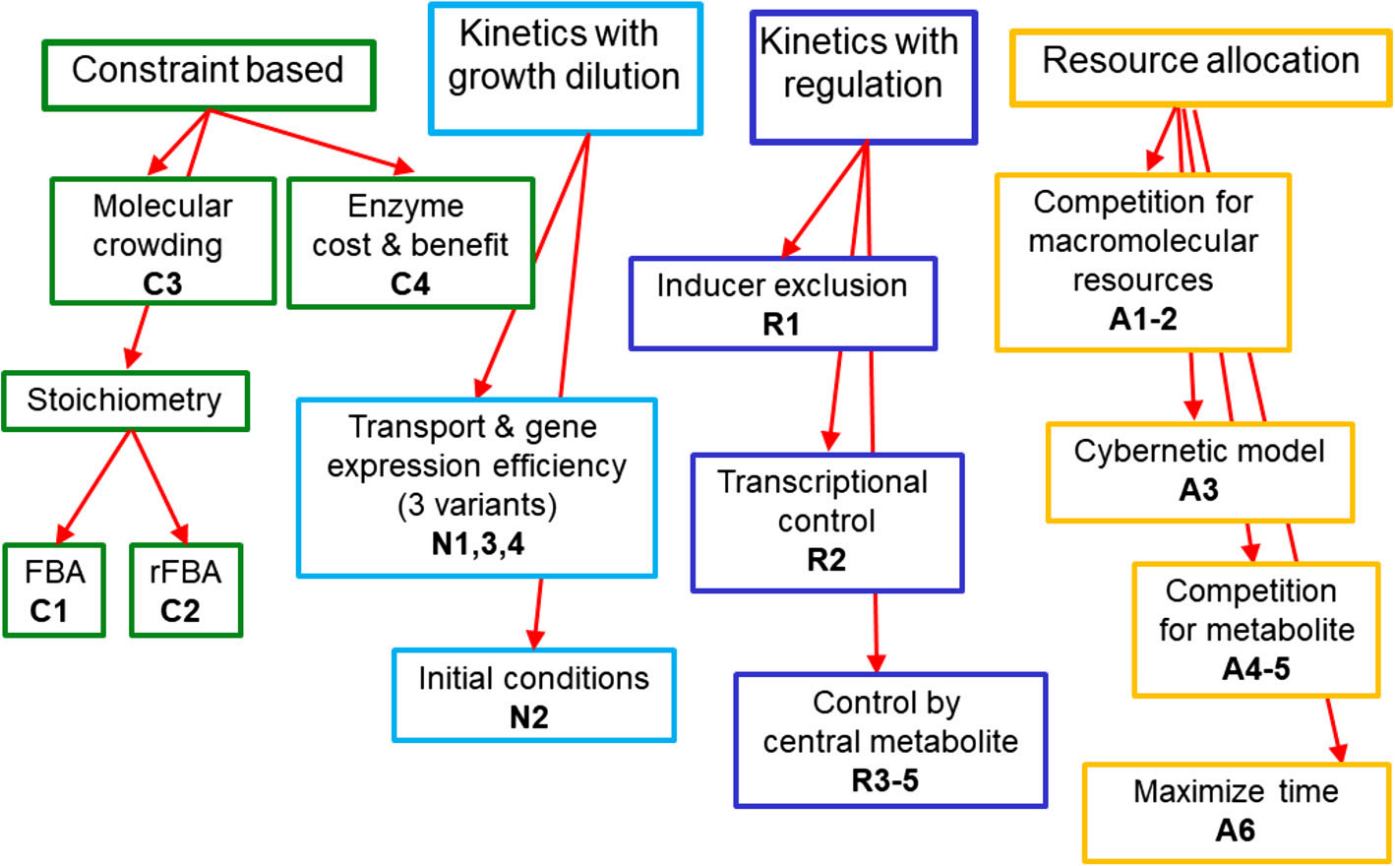
Overview of all model variants in the ensemble divided into four groups: constraint-based models, kinetic models with growth dilution, kinetic models with regulatory mechanisms, and resource allocation models

The models in the ensemble can be categorized according to regulatory, stoichiometric, and physiological constraints and differ from each other on only a single aspect. We distinguish four groups of models: (1) flux balance models that only define reaction kinetics for substrate uptake and by-product excretion, (2) kinetic models including the effect of growth dilution, (3) kinetic models with regulation on the metabolic and/or genetic level, and (4) resource allocation models. Figure 3 gives an overview of all model variants considered here. To quantify the output of the models and to allow a comparison of the model variants, the diauxic growth index *d* is introduced, indicating the degree of sequential utilization of the two carbon sources.

In order to further assess the performance of the models, we analysed two new experimental conditions, namely a glucose pulse applied to a culture growing on minimal medium with lactose and a batch culture with unequal initial conditions for the transport systems. In the latter case, the less preferred substrate, lactose, is used in the pre-culture and therefore, the respective enzymes are abundant at the beginning of the experiment. By comparing experimental data for these two conditions with model predictions, a number of models could be excluded, while other model variants are still not discriminable.

Based on the analysis, we conclude that models including known regulatory mechanisms like inducer exclusion and activation of the expression of transporters and enzymes by a global transcription factor [1] are best capable of accounting for the different experiments. It is likely, however, that a precise quantitative explanation of the control of the uptake and metabolization of carbohydrates involves a superposition of several different molecular mechanisms, acting on different time-scales. The precise contribution of each individual mechanism during a specific stimulation of the system remains to be determined.

## Methods

### Description of model structure: kinetic model

**Fig. 4:**
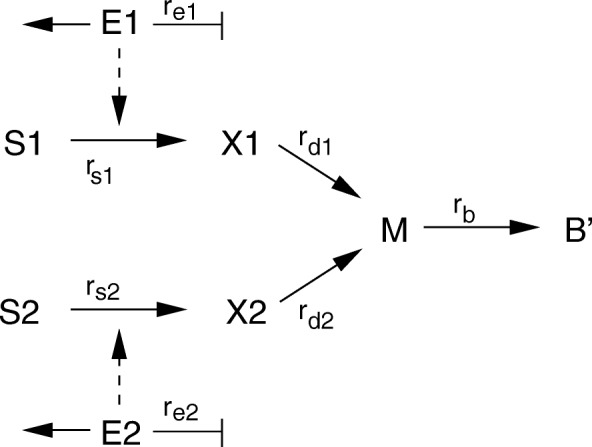
Basic reaction scheme for the diauxic growth network. Shown are all components, reaction rates (solid arrows), and catalytic activities and regulatory interactions (broken arrows). For the macromolecules, dilution by growth is considered in the model equations

All models of CCR included in the ensemble are variants of a simple model structure, based on the reaction scheme shown in Fig. 4. The models include both extracellular and intracellular components, and they are based on an explicit mass balance (because only in this way mass conservation can be assured). Since the components considered have a large number of molecules, a deterministic approach is followed. The mass balances are converted to a system of ordinary differential equations (ODEs), where each state variable represents the concentration of an extracellular or intracellular quantity. However, since different references are used when making this conversion, the resulting ODEs are different for intracellular and extracellular concentrations: for components in the bioreactor, the volume of the liquid inside the reactor is used, while for intracellular components the biomass is an adequate reference [7]. Converting mass balance equations to equations for concentration variables gives rise to dilution terms. In case of intracellular components with high internal fluxes, growth dilution can be neglected since this term contributes only marginally to the solution of the ODE.

The rationale behind the choice of the model structure schematized in Fig. 4 is as follows: in the extracellular space, the bioreactor, nutrients are provided that allow the biomass to grow. Therefore, the model takes into account two substrates with concentrations *S*_1_ and *S*_2_ and the entire biomass with concentration *B*. Each extracellular substrate is taken up by a transport reaction *r*_*si*_ and converted into intracellular components. Units used are *g*/*l* for extracellular substrates and biomass concentrations, *mol*/*gDW* for concentrations of intracellular components, and *mol*/*gDWh* for rates. Biomass is growing with the specific growth rate *μ* (unit 1/*h*). The ODEs for substrate and biomass are valid for all models:

1 $$\begin{array}{@{}rcl@{}} \dot{S}_{i} &=& -r_{si} \,\, w_{i}\,\, B, \quad i=1,2,  \end{array} $$

2 $$\begin{array}{@{}rcl@{}} \dot{B} &=& \mu \,\, B,  \end{array} $$

with molecular weight *w*_*i*_ of the substrates (rates *r* are given on a molar basis, while the medium concentration is given on a gram basis). The initial concentrations of the substrates and the biomass are denoted by $S_{i}(0)=S_{i}^{0}$ and *B*(0)=*B*^0^, respectively.

Substrates are converted to *X*_1_ and *X*_2_, representing small intracellular metabolites that in the case of lactose also act as inducers of the transporter proteins (local control). Dilution by growth is neglected for *X*_1_ and *X*_2_, since the rates *r*_*si*_ are high in comparison with the growth dilution term *μ* *X*_*i*_. Transport reactions, also including processes such as group translocation in case of a phosphorelay system [1], are under the control of enzymes *E*_1_ and *E*_2_. Both enzymes are synthesized; however, since proteins are stable, a degradation process is not explicitly taken into account, but only dilution by growth:

3 $$\begin{array}{@{}rcl@{}} \dot{X}_{i} &=& r_{si} \,\, - \,\, r_{di},  \end{array} $$

4 $$\begin{array}{@{}rcl@{}} \dot{E}_{i} &=& r_{ei} \,\, - \,\, \mu \,\, E_{i}.  \end{array} $$

The initial concentrations of the enzymes are denoted by $E_{i}(0)=E_{i}^{0}$.

Carbohydrates feed into central metabolic pathways such as glycolysis, the pentose phosphate pathway, and the tricarbon acid cycle. These pathways are represented by the intermediate metabolite *M*, for which we introduce the following equation:

5 $$\begin{array}{@{}rcl@{}} \dot{M} &=& r_{d1} \,\, + \,\, r_{d2} - r_{b}.  \end{array} $$

Since metabolites such as PEP, pyruvate, or fructose 1,6-bisphosphate are important messengers for metabolic fluxes, for example in E. coli [8], metabolite *M* is necessary for describing known basic regulatory schemes (global control).

Finally, component *B*^′^ is representative of the main biomass compartment, consisting of macromolecular species like protein, RNA, DNA, lipid, *etc.* Component *B*^′^ has a central role since it represents the resources of the cell. Resources are needed to synthesize enzymes that are involved in metabolic reactions, or other proteins. The component is represented by the following ODE:

6 $$\begin{array}{@{}rcl@{}} \dot{B^{\prime}} &=& r_{b} \,\, - \,\, \mu \,\, B^{\prime} \,,  \end{array} $$

with rate of synthesis *r*_*b*_ and a growth dilution term *μ* *B*^′^. The utilization of resources for enzyme synthesis is not included in the basic model structure, but explicitly added in the resource allocation models.

Special attention needs to be given to the choice of the growth rate *μ*. The growth rate of a population growing on a single substrate is related to the uptake rate and the yield. For a mixture of substrates, more sophisticated approaches can be found in literature ([9] and references therein). Since in our case, the focus is on the sequential uptake of substrates, in the kinetic models we combine both uptake rates in a simple equation, using yield coefficients *Y*_*i*_ as weighting factors:

7 $$\begin{array}{@{}rcl@{}} \mu &=& Y_{1} \,\, r_{s1} \,\, + \,\, Y_{2} \,\, r_{s2} \,.  \end{array} $$

The yield coefficients have the unit *gDW*/*mol*.

Kinetic rate laws determine the velocity of substrate conversion depending on the concentrations of the reactants. Modelling intracellular networks requires in addition terms that take into account metabolic and genetic regulation and signalling processes. The proposed scheme contains five metabolic reactions (*r*_*s*1_,*r*_*s*2_,*r*_*d*1_,*r*_*d*2_,*r*_*b*_) and, in addition for each enzyme, a term for synthesis (*r*_*e*1_,*r*_*e*2_) and a term for growth dilution (*μ* *e*_1_, *μ* *e*_2_). In the basic model structure, the kinetic rate laws are defined as follows:

8 $$\begin{array}{@{}rcl@{}} r_{s1} \,\, =& \,\, k_{s1} \,\,E_{1} \,\, \frac{S_{1}}{K_{1} \, + \, S_{1}} \,{,}&\quad r_{s2} \,\,= \,\, k_{s2} \,\,E_{2} \,\, \frac{S_{2}}{K_{2} \, + \, S_{2}}\,{,}  \\ r_{e1} \,\,=&\,\, k_{1} \,\, f_{1}\,{,}&\quad r_{e2} \,\,=\,\, k_{2} \,\, f_{2} \,{,} \\ r_{d1} \,\,=&\,\, k_{x1} \,\,X_{1} \,{,}&\quad r_{d2} \,\,=\,\, k_{x2} \,\,X_{2} \,{,}  \\ r_{b} \,\,=&\,\, k_{m} \,\,M\, {,}  \end{array} $$

Functions *f*_1_ and *f*_2_ are set to 1 here, but are further developed in models with regulation to take into account transcriptional control of enzyme synthesis.

Detailed kinetic modelling is often hampered by the difficulty of determining parameter values in an experimental set-up. The scaling of equations is an appropriate method for reducing the number of parameters and bringing the system into a defined time window [10]. As shown in Additional file 1, the equation system can be reduced in the number of parameters by an appropriate choice of scaling factors. For our model, extracellular substrates are scaled on the Michaelis-Menten constants *K*_*i*_ of the uptake rate, while biomass is scaled with respect to the Michaelis-Menten constants *K*_1_ and the yield coefficient *Y*_1_ for the first substrate. In the scaling of all intracellular components, the yield coefficient for the first substrate is present. In this way, the overall systems can be written with new parameters that take into account the ratio of the yield coefficients, the ratio of certain velocity constants, and the ratio of the molecular weight of the substrates. The equations of the rescaled model structure are given in Additional file 1: Section A.1.

Other approaches to reduce the number of state variables and parameters are based on the (quasi-)steady-state assumption for intracellular metabolites [11]. Using the fact that reaction rates are explicit functions of the intracellular metabolites, the quasi-steady-state assumption for the fast variables *X*_1_, *X*_2_, and *M* leads to a set of algebraic equations from which the fast variables (intracellular metabolite concentrations) can be solved as explicit functions of the slow variables (enzyme concentrations, external substrate concentrations) [12].

### Description of model structure: dynamic flux balance analysis model

Another use of the quasi-steady-state assumption does not use the rate equations, but combines metabolic flux analysis for the fast part of the system with kinetic modelling for the slow part. This requires the computation of intracellular fluxes (rates at steady state) directly from known uptake and excretion rates, without determining the concentrations of intracellular metabolites, and the use of these fluxes as inputs in the remaining ODEs. At (quasi-)steady state, the incoming and outgoing fluxes are equal for every intracellular metabolite and hence, the following constraint holds for rate vector $\underline {r}$:

9 $$\begin{array}{@{}rcl@{}} N \,\, \underline{r} \,\, = \,\, \underline{0}\, {,}  \end{array} $$

with *N* as the stoichiometric matrix. For the intracellular metabolic network of Fig. 4, we have:

10 $$\begin{array}{@{}rcl@{}} N \,\, = \,\, \left[\begin{array}{ccccc} 1 &-1 &0 &0 &0 \\ 0 & 0 &1 & -1 & 0 \\ 0 & 1 & 0 & 1 & -1 \end{array}\right] \, {,}  \end{array} $$

where the columns correspond to *r*_*s*1_, *r*_*d*1_, *r*_*s*2_, *r*_*d*2_, and *r*_*b*_, and the rows to *X*_1_, *X*_2_ and *M*, respectively.

In order to combine the algebraic equations for the part of the system that is at quasi-steady state with the ODEs for the slow variables, we rewrite Eq. (9) by making appropriate substitutions for the rates of the uptake reactions defined in Eq. (8):

11 $$\begin{array}{@{}rcl@{}} N^{\prime} \,\, \underline{x} \,\, = \,\, \left[ \begin{array}{ccccc} h(S_{1}) & -1 & 0 & 0 & 0 \\ 0 & 0 & h(S_{2}) & -1& 0 \\ 0 & 1 & 0 & 1 & -1 \end{array}\right] \,\, \left(\begin{array}{c} E_{1} \\ r_{d1} \\ E_{2} \\ r_{d2} \\ r_{b} \end{array}\right) \,\, = \,\, \underline{0},  \end{array} $$

where *h*(*S*_1_)=*k*_*s*1_ *S*_1_/(*K*_1_ + *S*_1_) and *h*(*S*_2_) = *k*_*s*2_ *S*_2_/(*K*_2_ + *S*_2_).

Since the stoichiometric matrix has more columns (unknown rates) than rows (mass balance equations), as is generally the case, $ N^{\prime } \,\, \underline {x} \, = \, 0$ does not have a unique solution. Constraints on the upper and lower bounds of fluxes and additional constraints can be provided to reduce the space of solutions, and an objective function added to select those solutions that optimize a specified criterion. This methodology is called Flux Balance analysis (FBA) [6] and the respective equations are given by:

12 $$\begin{array}{@{}rcl@{}} \max && \quad \underline{c}^{T} \,\, \underline{x}\,,  \\ &\text{s.t.}& N^{\prime} \,\, \underline{x} \,\, = \,\, \underline{0}\, {,}  \\ && H \,\, \underline{x} \,\, \le \,\, \underline{h}\, {,}  \end{array} $$

with a matrix *H* taking into account additional constraints and $\underline {h}$ a vector of numerical bounds. We choose $\underline {c}$ such that $\underline {c}^{T} \,\, \underline {x} = r_{b}$, in other words, the criterion to maximize is the rate of accumulation of the main biomass component *B*^′^. This rate is set equal to *μ*.

The above linear optimisation problem can be solved at every time point and the algebraic solution provided as input to the ODE system for the slow variables, more precisely Eqs. (1), (2), and (4) for the concentrations of substrates, enzymes, and biomass. This approach is known as dynamic FBA in the literature [5].

### Numerical simulation, parameter estimation, and cluster analysis

All simulations were performed with Matlab and all files can be found here:

https://sourceforge.net/projects/diauxic-growth-model-ensemble/

The dynamic optimization programs in some of the resource allocation models were solved by means of DOTcvp [13].

The rescaled models have only a small number of parameters. Moreover, some of these parameters – like the ratio of the molecular weights or the ratio of the yield coefficients – are known from batch experiments with single substrates [14]. For model C3 (crowding), for example, only three free parameters, the two crowding coefficients and the upper limit on membrane space, need to be determined. These remaining parameter values for the models were selected in two different ways. First, for each model, parameters were estimated using a stochastic search algorithm that maximizes the value of *d* (diauxic growth index). Second, for some model variants, parameters were estimated to reproduce time-course data from a batch experiment with *E. coli* growing on glucose and lactose. Data were taken from [14] and the model fits were shown in [2] but were not documented there. A least-square problem was formulated to minimize the differences between simulation and experimental data. A simple genetic algorithm in Matlab [15] was used to find the best-fit parameters, setting the population size to 40 and the number of generations between 30 and 40 (see also Additional file 1). Since the number of parameters to be estimated is low, in both optimization problems the algorithms search nearly the complete parameter space and converge quickly.

Simulations of the model with estimated parameters can be found here:

https://sourceforge.net/projects/diauxic-growth-model-ensemble/

For the hierarchical clustering of simulated time-courses, the Euclidian distance between the time-courses was determined and the inner squared distance between two clusters was calculated by means of the Matlab routine *linkage* (with *ward* and *euclidean* as parameters). For each cluster, the mean value of the time-courses at each time point was calculated as representative of the cluster. The maximal number of clusters was predefined in the Matlab routine *cluster*.

## Results

### Ensemble of CCR models

The models in the ensemble can be grouped into four categories, characterized by different types of reaction mechanisms and control structures. As a thought experiment, consider the basic kinetic model structure of Eqs. (1)–(7), discussed in the “Methods” section, when there is a perfect symmetry between the availability, uptake, and metabolization of *S*_1_ and *S*_2_, that is, *S*_1_(0)=*S*_2_(0), *r*_*s*1_=*r*_*s*2_, *r*_*d*1_=*r*_*d*2_, *r*_*e*1_=*r*_*e*2_, and *E*_1_(0)=*E*_2_(0). Obviously, in this case the time-courses of the variables will not exhibit diauxic growth. The same holds for the basic flux balance model structure of Eqs. (11)–(12).

The CCR models introduced below are all based on different single changes in the structure of the equations and/or the parameter values to break the symmetry between the pathways of the two substrates. For chosen parameter values, all model variants are capable of reproducing diauxic growth and growing on both substrates separately. The equations of the individual model variants, in particular the changes with respect to the basic model structures, are detailed in Additional file 1: Section A.2, together with a plot of the diauxic growth behaviour generated by the model.

**Fig. 5:**
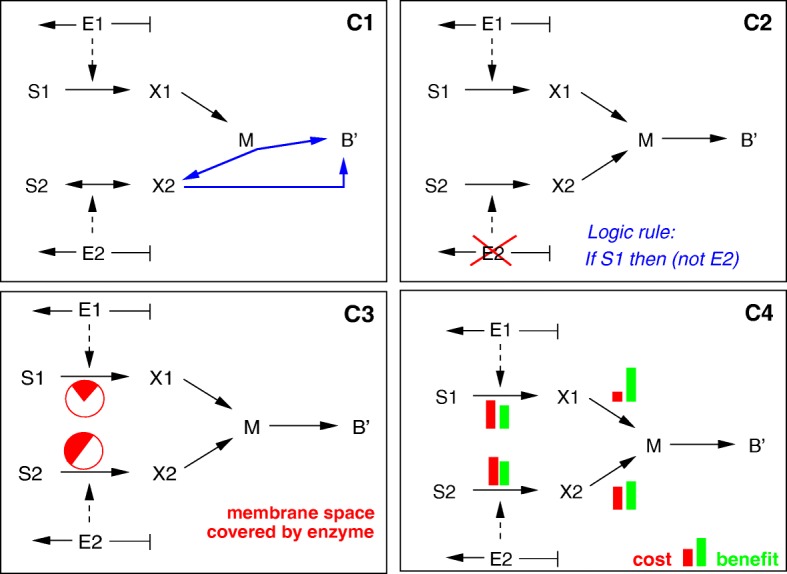
Reaction schemes for models in group 1 (constraint-based models). Upper left: FBA (**C1**), upper right: regulatory FBA (**C2**), lower left: molecular crowding and limits on membrane space (**C3**), lower right: costs and benefits of enzyme production (**C2**)

Group 1 consists of so-called constraint-based models and are variants of the flux balance analysis model structure of Eqs. (11)–(12). A first way to break the symmetry of the uptake pathways in this model structure is to slighty modify the stoichiometric matrix (model **C1**). Motivated by the diauxic growth behaviour observed with glucose and acetate [16], we modified reaction *r*_*b*_ in Fig. 4. In contrast to the reaction scheme of Fig. 4, in model C1 metabolite *M* is a central metabolite for substrate *S*_1_ only. From *M*, macromolecules are produced, but at the same time *X*_2_ as a side-product. Metabolite *X*_2_ can be excreted or converted directly into macromolecules (but inefficiently, at a lower maximal rate than *X*_1_). In the first growth phase, substrate *S*_1_ is consumed and, assuming the uptake reaction is reversible and the inefficient biomass reaction is inactive, substrate *S*_2_ is produced. In the second growth phase, only *S*_2_ is taken up and converted into biomass. The modified scheme is shown in Fig. 5.

A regulatory component in the original model set-up is also sufficient to break the symmetry. Regulatory interactions can be easily incorporated into the framework of FBA (**C2**) by means of a set of rules in Boolean logic (regulatory FBA or rFBA, [17]). These logic rules relate an environmental variable (e.g., the availability of a substrate) to the presence or absence of enzymes (and thus their corresponding reaction rates) in the model. The rules are executed in a sequential manner resulting in a modified stoichiometric matrix. In model C2, diauxic growth is obtained by a single rule inactivating enzyme *E*_2_, and thus the uptake of *S*_2_, in case substrate *S*_1_ is available (Fig. 5).

An interesting extension of FBA takes into account the limited space available for transporters in the membrane, and for enzymes in the cytoplasm more generally, an effect called molecular crowding [18, 19]. Imposing a membrane space constraint results in an additional linear inequality in the constraint-based model (**C3** in Fig. 5). Assuming that *E*_1_ and *E*_2_ occupy different amounts of membrane space is sufficient for breaking the symmetry between the two substrates and for diauxic growth behaviour to occur.

A number of recent studies emphasized the characterization of enzyme production in terms of costs (ATP and substrate usage) and benefits (generating energy and precursors for biomass) [20, 21]. These ideas are considered in a further model variant based on FBA: for both pathways, from the uptake of *S*_1_ and *S*_2_ to central metabolism, the trade-off between the cost of making the enzymes and the benefits gained by their use in cellular metabolism leads to an additional inequality in the model (**C4** in Fig. 5). The inequality expresses that the costs of enzyme synthesis should not exceed the benefits by a specified maximum amount.

Group 2 consists of mathematical models that include kinetic terms for enzymatic reactions and enzyme synthesis. The dynamics of the underlying biochemical reaction systems have a crucial property: for state variables there is usually a balance between reactions producing and consuming a component. In addition, what is overlooked in many cases, a dilution term appears in ODEs for concentration variables describing the dilution of enzymes and metabolites due to growth of the population [22]. The dilution term arises from the choice of biomass as the reference volume of the intracellular state variables. As a consequence, the concentration of enzymes and metabolites decays if the rate of growth dilution exceeds the rate of synthesis.

**Fig. 6:**
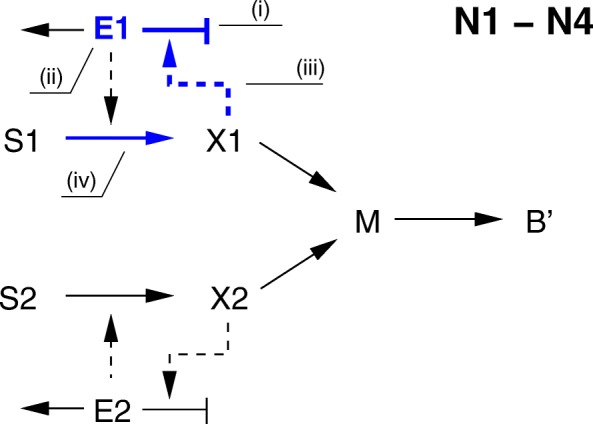
Reaction schemes for models in group 2 (kinetics-based models with growth dilution). The models include induction of enzyme synthesis by the metabolites *X*_1_ and *X*_2_. In blue are shown different ways in which the dynamics of enzyme *E*_1_ can be modified, in every case leading to diauxic growth: (i) the maximum rate of enzyme synthesis (**N1**), (ii) the initial enzyme concentration (**N2**), (iii) the affinity of the inducer for its transcription factor and thus the effect on enzyme synthesis (**N3**), or (iv) the maximum rate of substrate uptake (**N4**)

Dilution effects may give rise to diauxic growth. In order to see this, consider kinetic models extended with an additional regulatory interaction, the activation of enzyme synthesis by the metabolites *X*_1_ and *X*_2_ (Fig. 6). The symmetry between the two carbon sources can be broken by changing the values for (i) the maximum rate of enzyme synthesis (**N1**), (ii) the initial enzyme concentration (**N2**), (iii) the affinity of the inducer for its transcription factor and thus the effect on enzyme synthesis (**N3**), or (iv) the maximum rate of substrate uptake (**N4**). For the chosen parameter values the enzymes of the preferred substrate will accumulate, whereas those of the non-preferred substrate will dilute out.

Models in group 3 extend the kinetic expressions used so far by including additional regulatory mechanisms. The most prominent regulatory effects described in the literature on *E. coli* are inducer exclusion and activation by a global transcription factor. Inducer exclusion means that a transport enzyme (e.g., for lactose uptake) or an enzyme involved in metabolism of the substrate (e.g., glycerol metabolism) is subject to control [1]. In the case of lactose and glycerol, a component of the glucose transport system (PEP-dependent phosphotransferase system, PTS), the protein EIIA, acts as an inhibitor of the enzymes. Metabolic regulation of the activity level of the enzymes is very fast in comparison to gene expression regulation. In the case of the glucose-lactose diauxie, a second mechanism based on transcription regulation has been described to control the enzyme concentration: cAMP, a small molecule that is synthesized when the glycolytic flux is low, acts as an activator of the global transcription factor Crp. Crp is involved in a number of cellular processes and most prominently in the control of nearly all carbohydrate uptake systems.

**Fig. 7:**
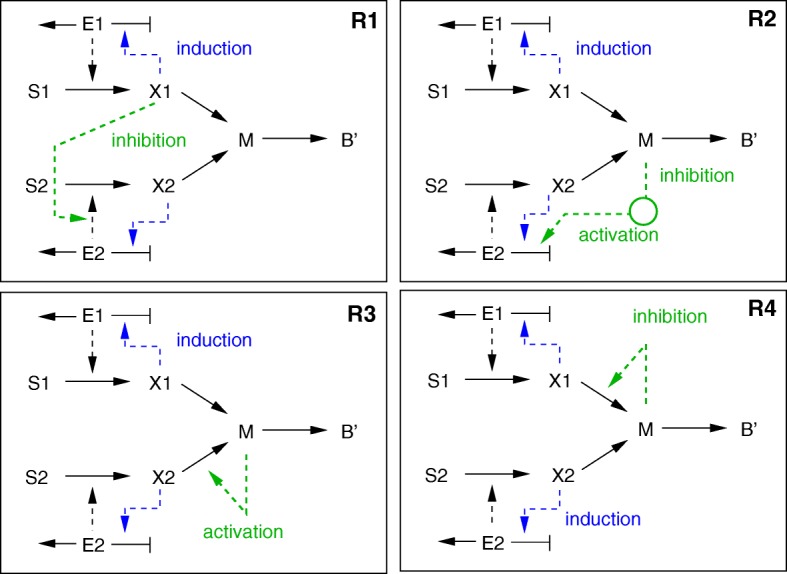
Reaction schemes for models in group 3 (kinetics-based models with regulation). Upper left: inducer exclusion (**R1**), upper right: activation by a global transcription factor, itself inhibited by a central metabolite (**R2**), lower row: model variants with a central metabolite modifying the inducer concentrations (**R3** and **R4**)

Within our simplified scheme, the two mechanisms are represented as follows (Fig. 7). In the case of inducer exclusion (**R1**), metabolite *X*_1_ acts as a regulatory metabolite and inhibits enzyme *E*_2_ responsible for uptake of substrate *S*_2_. Activation by a transcription factor is a two-step process: a lower flux through central metabolic pathways will lead to lower concentrations of the metabolites involved, in particular the central metabolite *M*. The lower concentration of *M* leads to the synthesis of a second messenger like cAMP and subsequent activation of the transcription factor. Since the second messenger and the transcription factor are not explicitly represented in the model, metabolite *M* is assumed to directly act as an inhibitor of the synthesis of *E*_2_ (**R2**). Choosing numerical values for strong inhibition by inducer inclusion alone, or for strong activation by a global transcription factor alone, is sufficient to obtain diauxic growth behaviour. Although in both model variants induction of the transport system by *X*_1_ and *X*_2_ is assumed as well, like in the models N1-N4, this addition is not necessary for diauxic growth.

The central metabolite *M* offers further possibilities for control schemes. As described for *E. coli*, the metabolite fructose 1,6-bisphosphate is directly involved in the activation and/or repression of transcription factor Cra (FruR) [1]. Cra is involved in the control of gluconeogenesis. This regulatory mechanism is adapted to our modelling framework in two different ways. First, *M* can act as an activator of the consumption of metabolite *X*_2_ (**R3**). As a consequence, for a higher concentration of *M*, the concentration of *X*_2_ will be lower, which decreases its ability to induce the uptake system of *S*_2_. Alternatively, *M* can act as an inhibitor of the consumption of metabolite *X*_1_ (**R4**). In this way, for a higher concentration of *M*, the concentration of *X*_1_ will be higher as well, which increases its ability to induce the uptake system of the preferred substrate *S*_1_. For both schemes, induction of the transport systems by *X*_1_ and *X*_2_ is necessary.

**Fig. 8:**
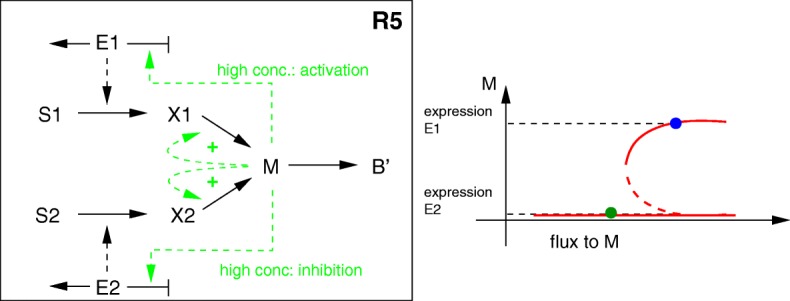
Additional reaction scheme for models in group 3 (kinetics-based models with regulation). Left: the reactions producing *M* are activated by *M* itself (product activation), in addition to the control of enzyme synthesis by *M* (**R5**). Right: depending on the incoming flux to *M*, two stable steady states are possible, as shown in the bifurcation diagram. Solid red curves denote stable steady states, while the dashed red curve denotes the unstable steady state. The circles mark the steady states at the beginning (blue) and at the end of the simulation (green)

An additional control scheme can be derived from the following line of reasoning (which we could not relate to any known regulatory mechanism in bacterial cells). Assume again that metabolite *M* controls the synthesis of the two enzymes, but in a different manner: high concentrations of *M* lead to synthesis of *E*_1_, whereas low concentrations lead to synthesis of *E*_2_. If the total incoming flux to *M* and the concentration of this metabolite are positively correlated, only the first substrate will be consumed because only the first enzyme is synthesized. However, if more complex kinetic expressions for the reaction producing *M* are used, such as product activation, multiple steady states may appear (**R5**). This is illustrated by the bifurcation diagram in Fig. 8: the kinetic rate law leads to two stable steady states for *M* depending on the incoming flux. Assuming that the starting condition is such that only enzyme *E*_1_ is synthesized, the first substrate is taken up (blue circle). As the concentration of *S*_1_ decreases, the incoming flux decreases as well and the system moves along the upper stable branch to the left until the bifurcation point is reached, where it drops to the lower stable branch. This leads to an increase in the amount of enzyme *E*_2_ and the consequent uptake of *S*_2_. However, the flux to *M* is lower in the second growth phase (green circle). Note, that for bringing the system back to the first steady state, a disturbance directly increasing *M* is necessary (it is not sufficient to feed substrate *S*_1_ again).

Group 4 comprises models that distribute cellular resources over the network components. Resource allocation can be integrated into the models in two different ways, by the solution of an optimization problem or by the definition of specific regulatory interactions influencing the kinetics of the reactions. Models in both subgroups can be seen as variants of the basic kinetic model structure, but models in the first subgroup integrate elements of the flux balance model structure as well, in particular the determination of the value of some of the variables by maximizing an objective function.

**Fig. 9:**
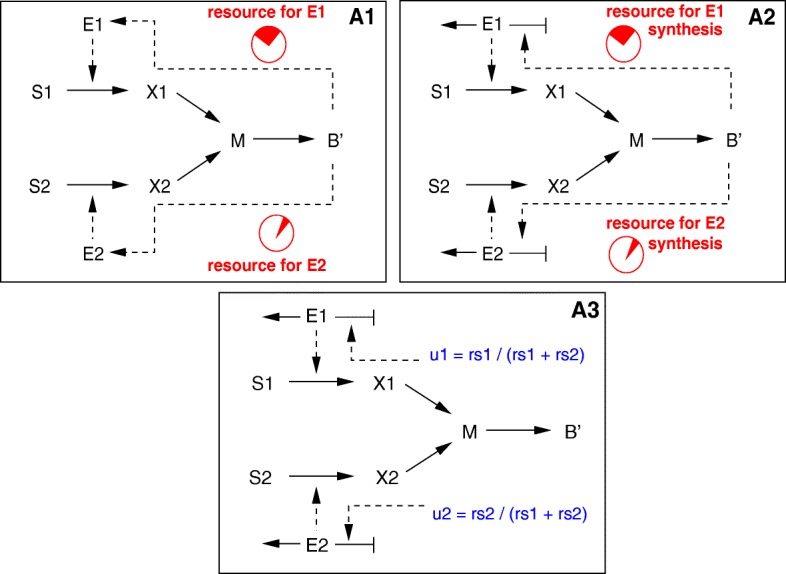
Reaction schemes for models in group 4 (resource-allocation-based models). Upper row: static (left) and dynamic (right) optimization for resource allocation (**A1** and **A2**). Lower plot: cybernetic modelling approach (**A3**). The rates of enzyme synthesis are set in proportion to the fractional uptake rates *u*_1_, *u*_2_ of the substrates

For the first subgroup of resource allocation models an objective function is defined, corresponding to either the maximization of the incoming fluxes or the maximization of biomass over a time-interval running from 0 to *t*_*end*_. In both cases, the resource to be allocated consists of the intracellular compartment *B*^′^ (Fig. 9). In the first model variant (**A1**), a fraction of *B*^′^ is available for the two transport systems; that is, the (weighted) sum of the concentrations of both enzymes is limited by an upper bound smaller than *B*^′^. Like for the other model variants in the ensemble, the symmetry can be broken (and substrate *S*_1_ taken up preferentially) by a suitable choice of the weights and other parameters.

The optimization problem for model A1 is static, like in dynamic FBA, in the sense that it is formulated at a specific time-point, for specific concentrations of substrates, biomass, *etc.* The result of the optimization problem (the enzyme concentrations) is fed into the ODEs governing the dynamics of the other variables. The second model variant (**A2**) involves a fully dynamic optimization problem. In this model the rates of enzyme synthesis *r*_*ei*_, rather than the concentrations *E*_*i*_, depend on *B*^′^, and the drain of resources towards enzyme synthesis is explicitly accounted for in the ODE for *B*^′^, by including a reaction with rate *u*_*max*_ *B*^′^, where *u*_*max*_ is the fraction of the biomass component utilized for protein synthesis. The resource is dynamically distributed over the enzymes so as to maximize the biomass produced at *t*_*end*_. In addition, we require the size of the internal pool of *M* to be smaller than a certain maximum value. Setting a limit for *M* has a strong influence on the system dynamics in that, if the parameter values for the two uptake pathways are different, it breaks the symmetry between the two pathways and causes one substrate to be preferred.

Cybernetic modelling was introduced some decades ago to describe the behaviour of a population exhibiting diauxic growth [23, 24]. The different variants of cybernetic modelling have notably been capable of accounting for a variety of scenarios for simultaneous or preferential uptake of carbon sources in *E. coli* [23, 25]. Cybernetic models provide a coarse-grained description of microbial kinetics and allocate resources to the synthesis or the activity of specific enzymes in proportion to their return, that is, the growth rate or the quantity of substrate metabolized by the enzyme. They provide two handles for resource allocation: control of enzyme activity and control of enzyme synthesis [23, 24]. Our adaptation of cybernetic modelling, shown in Fig. 9, is restricted to the latter option and accordingly modifies the rate expressions for enzyme synthesis (**A3**). In order to break the symmetry in this case, the maximum uptake rate for the two enzymes was set to different values.

**Fig. 10:**
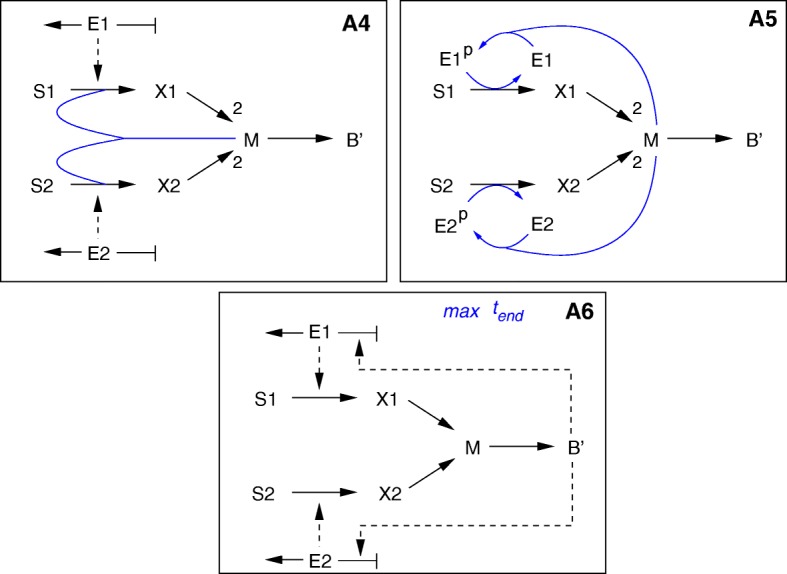
Reaction schemes for models in group 4 (resource-allocation-based models). Continued. Upper row: competition for *M*, the energy carrier used in the transport reactions (**A4**) and PTS competition model (**A5**). Lower row: maximization of lifespan (**A6**)

Finally, we consider model variants that distribute energy for transport over the two transport systems. This can be realized in two ways, as schematically shown in Fig. 10. In the first variant, metabolite *M* is needed directly for transport and its concentration is included in the transport kinetics. Metabolite *M* here represents an energy carrier like ATP. The ODE for *M* is updated accordingly to take into account this additional mass flow, and the stoichiometric coefficient for the production of *M* from *X*_*i*_ is changed to 2 (**A4**). The second variant mimics the observation that group translocation processes transfer phosphoryl groups from one protein to another, as for the above-mentioned PTS (**A5**). In particular, metabolite *M* can be seen as a proxy for PEP, that is, the energy source for glucose transport in *E. coli* and other bacteria, whereas the PTS is represented by the protein *E*_1_. The enzymes occur in a free (unphosphorylated) and a phosphorylated form to mimick energy consumption by the transport reactions.

A long time ago already it was suggested that cellular systems tend to extend their lifespan for as long as possible ([26], analysed also in [27]). In case a number of substrates are available, this principle would automatically result in the sequential uptake of the substrates, because it would maximize the lifespan *t*_*end*_ of the microorganism. In the model variant **A6** we therefore define an additional lifespan variable and maximize this variable by means of the so-called free time optimal control approach [27]. In order to guarantee that the cells remain alive, we add a minimal value for *B*^′^ as a constraint for the optimization process. Again, the resources for synthesizing the enzymes are provided by compartment *B*^′^ (see Fig. 10 for the reaction scheme). Interestingly, no further structural modifications or changes in kinetic parameter were necessary to obtain diauxic growth behaviour for this model variant.

### Diauxic growth index

Every model variant in the ensemble presented above is capable of generating diauxic growth behaviour for chosen parameter values. The different (rescaled) models share parameters such as yield coefficients for substrate uptake (Additional file 1: Section A.1). However, some of the parameters are unique for a model variant and determine its specificity (on average, there are 2 to 3 kinetic parameters to choose for each model). One could ask whether this model-specific set of parameters can be tuned in such a way that - possibly - a nearly “perfect” diauxic growth behaviour is obtained. In order to answer this question, we turn the intuitive notion of “perfect” diauxic growth into a quantitative measure, by defining the diauxic growth index, *d*. The index varies between 0 (no diauxic growth, that is, parallel uptake of both substrates) and 1 (perfect sequential uptake). More precisely, *d* is defined as the (absolute) difference of both uptake rates multiplied by the biomass, integrated over the time-interval [0,*t*_*end*_]:

13 $$ d \,\, = \,\, \frac{1}{2} \,\,\, {\int \limits_{t=0}^{t_{end}}} \,\, |r_{1}(t) \,\, - \,\, r_{2}(t) | \,\, b\,\, dt \, {.}   $$

To motivate the definition of *d*, consider the initial value *s*_0_=1 unit for a single substrate. With a yield coefficient of 1, the integral for this substrate is ${\int } r \,\,b \, dt \,= \, s_{0} \, = \, 1$ (*r* is the uptake rate) when the substrate has been completely consumed by biomass *b*. For two substrates that are consumed one after the other, the value of *d* obtained from Eq. (13) must therefore be 1 and in the case of parallel consumption of the two substrates *d*=0.

**Fig. 11:**
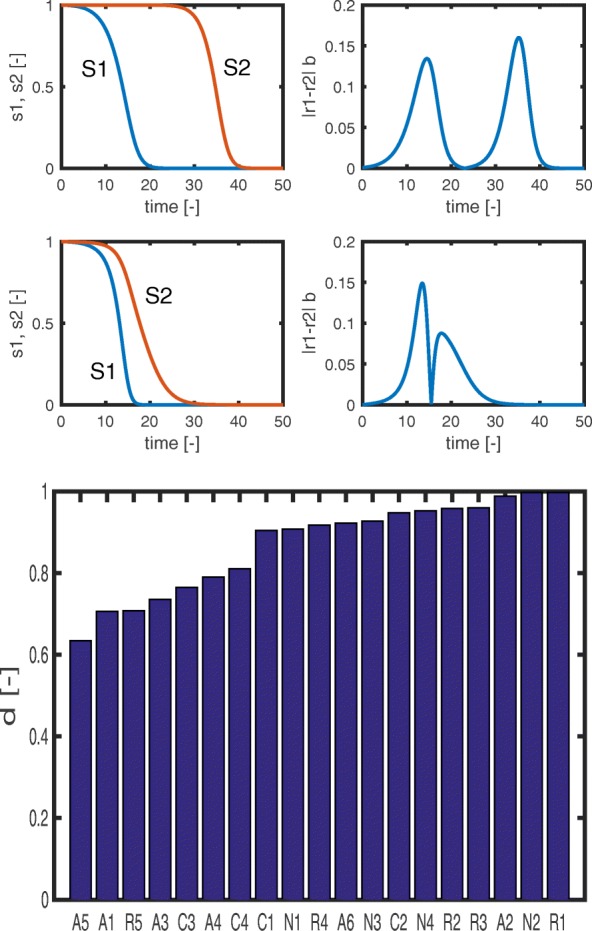
Upper plot: Examples of a high diauxic growth index, that is, perfect sequential growth on substrates *S*_1_ and *S*_2_ (upper row, model **R1**), and a low diauxic growth index (lower row, model **A5**). The plots show the time-course of the substrates and the time-course of the absolute difference of the uptake rates multiplied by the biomass, that is, the integrand of the diauxic growth index *d*. Bottom plot: Diauxic growth index for all model variants in the ensemble

For each model variant in the ensemble, we fine-tuned the model-specific parameters with a stochastic search algorithm (while keeping the same value for the shared parameters), so as to maximize the diauxic growth index. The number of model-specific parameters for each model is low since most parameters in the scaled model are given as ratios of two original parameters characterizing the pathways for the two substrates (for example, maximal uptake rates or yield coefficients). We set the scaled parameters to 1, so that the pathways for the two substrates have the same kinetic properties. This ensures that if diauxic growth occurs, it arises from the additional structural assumptions in the model variants that break the symmetry between the pathways. In model R1 (inducer exclusion), for example, only the inhibition constant *K*_*I*_ remains to be chosen. Figure 11 shows time course data for model variants R1 (nearly perfect diauxic growth) and A5 (partial co-consumption).

The sorted indices thus obtained are also shown in Fig. 11. As expected, most kinetic models with regulation have a high index score, but it can be seen in Fig. 11 that models belonging to other categories may also show nearly perfect diauxic growth, such as models N2 and A2.

### Comparison with experimental data

**Fig. 12:**
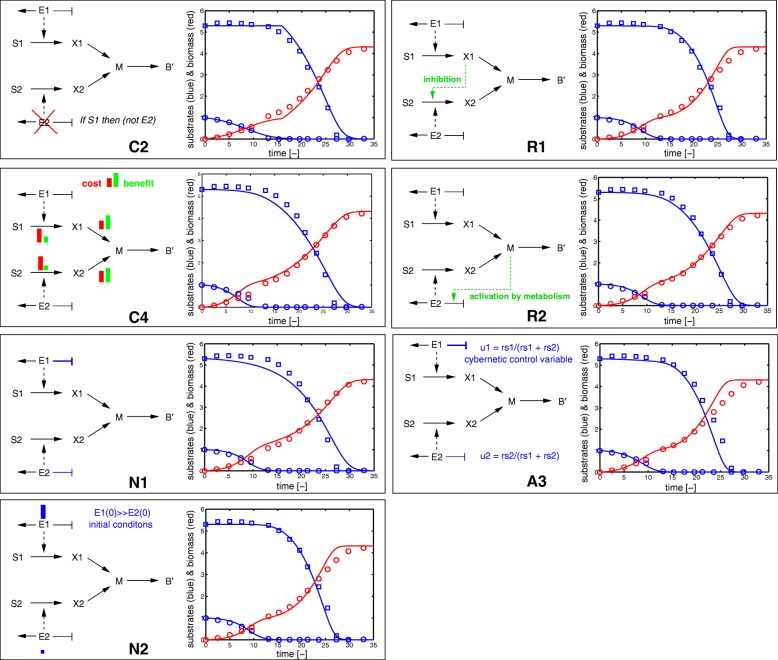
Comparison of the predictions of some selected model variants (solid lines) with experimental data [14] (open circles and squares) after parameter fitting. The substrates and biomass are shown in blue and red, respectively. More details on the fitting procedure can be found in the Additional file 1

As already shown in [2] (Fig. 3 therein), the predictions from the models in the ensemble can be directly compared with experimental data. We took data from a standard diauxic growth experiment with glucose and lactose as substrates for *E. coli* (Fig. 1) and adjusted the parameters so as to obtain a good fit. Figure 12 shows a selection of fitted models against experimental data (see also Additional file 1: Section A.4).

**Fig. 13:**
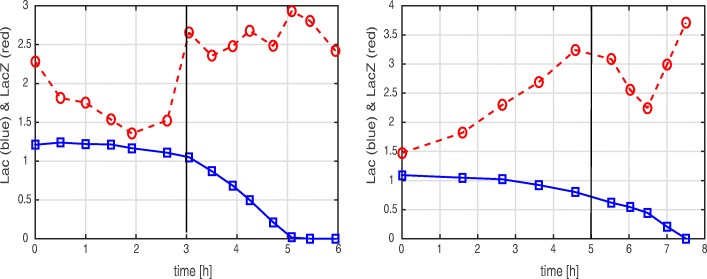
Time-course of lactose concentration (blue, solid line) and LacZ activity as proxy for the LacZ concentration (red, dashed line) for two experimental conditions. Left: a preculture in minimal medium with lactose is followed by a main culture in minimal medium with glucose. The dark vertical line indicates the timing of the depletion of glucose. Right: a glucose pulse experiment during exponential growth on minimal medium with lactose. The dark vertical line indicates the timing of the pulse. Data are taken from [14] (there: Supplementary file 2, Figures 6 (left) and 10 (right))

Since a large number of model variants show a satisfactory fit, additional experiments are needed for model discrimination. These experiments should stimulate the system in a different way than the reference batch experiment of Fig. 1. We chose two dynamic experiments from a large collection of experimental data [14]: first, a dynamic pulse experiment where the preferred substrate (glucose) is pulsed during exponential growth on a non-preferred second substrate (lactose); and second, an experiment in which an enzyme necessary for the assimilation of the non-preferred substrate (LacZ) has a high initial concentration at the beginning of the experiment (Fig. 13).

In order to discriminate between the model variants, we first simulated the reaction systems with the parameters used in Fig. 11 in the conditions of the new experiments. For the pulse experiment, the timing of the pulse was adapted for each model according to the growth rate; that is, for a model with a slower growth rate, the pulse was applied later. The models N2 and A2 were omitted from the analysis, since N2 imposes an initial condition conflicting with the conditions of the experiment with a high initial enzyme concentration, and the dynamic programming tool used for A2 [13] does not allow the occurrence of externally-determined pulses.

Second, we clustered the predicted time-courses by means of a hierarchical clustering algorithm with a Euclidian distance measure and a predetermined number of clusters. For each cluster, a representative time-course was computed by taking the mean value of the model predictions at every time-point. The resulting clusters were then qualitatively compared with the experimental data. In particular, the following qualitative characteristics were retained (Fig. 13). For the experiment with the high initial LacZ concentration, lactose is not taken up during growth on glucose and LacZ is diluted out. However, the expression of *lacZ* resumes after glucose depletion. In the glucose pulse experiment, lactose uptake slows down (slightly) after the pulse and LacZ synthesis stops, but both resume shortly afterwards. Data were rescaled for the timing of the pulse to allow a fair comparison between the models.

**Fig. 14:**
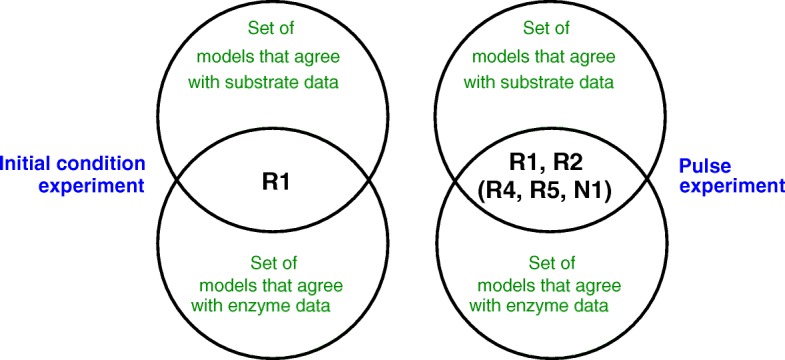
Left: intersection of the sets of models capable of qualitatively reproducing the substrate and/or the enzyme concentration time-courses observed in the initial condition experiment. Right: intersection of the sets of models capable of qualitatively reproducing the substrate and/or the enzyme concentration time-courses observed in the pulse experiment. The models in the intersection have been explicitly indicated (the results for the models between brackets are ambiguous, see main text)

All simulated time-courses for the individual models together with the mean time-courses of the clusters, as well as the dendrograms returned by the clustering procedure, are shown in Additional file 1: Section A.3. Since more than one measured state variable is available, the clustering procedure can be repeated for each measured state variable and the intersections between clusters give an indication of which model variants perform best. We illustrate the outcome of this (repeated) clustering procedure for the two experiments reported in Fig. 13. Inspection of the trend of the mean time-courses in a cluster allows one to determine if the models in the cluster are able to qualitatively reproduce the above-mentioned characteristics of the experiments. For each experiment, this results in a list of models whose predictions match the time-course of the substrate, the time-course of the enzyme, or both (Fig. 14).

For the experiment with the high initial enzyme concentration, most models fail to reproduce the characteristic observation that lactose is not taken up although the necessary enzyme is highly expressed. The only model succeeding in accounting for both the substrate and enzyme time-courses is R1, the inducer exclusion model (Fig. 14). For the pulse experiment, the results are somewhat ambiguous. A number of models reproduce the substrate time-courses well. What poses a problem, however, is that lactose uptake slows down after the glucose pulse for a short time only, simultaneously with an equally-short decrease of the LacZ concentration. Since the LacZ concentration and lactose uptake both resume after this short period, a number of models are - in principle - able to account for the experiment. As indicated in Fig. 14, models with inducer exclusion (R1) and with global transcription factor activation (R2) are in the intersection, but also models that take into account control by central metabolite M (R4) or a different rate constant for gene expression (N1) are consistent with this experiment.

## Discussion and conclusions

Carbon catabolite repression has been under investigation since a long period of time. For applications in biotechnology, for example, the understanding of the interplay between different carbohydrate uptake systems is of crucial importance. Gaining a better comprehension of their interactions allows modification in such a way that both substrates are taken up in parallel rather than sequentially [28, 29].

Ensemble modelling is an advanced method for describing cellular systems in case one is confronted with large uncertainties. It allows different hypotheses to be formulated and analysed first from a theoretical point of view. Usually, when setting up a model ensemble, different kinetic parameters and/or network structures are explored to find models that best account for the available data and that can be used for making predictions in new experimental scenarios. In the ensemble proposed here, making predictions involves simulation but also solving an optimization problem for some of the model variants. The latter models are based on classical approaches, like flux balance analysis and its derivatives, but also include new schemes that distribute cellular resources over metabolic pathways.

Note that the model variants involving optimization provide explanations of diauxic growth that are not based on the causal effect of regulatory mechanisms, but on the assumption that microorganisms have evolved to optimize a criterion that confers them a selective advantage. The mechanisms underlying this supposedly optimal behaviour may be unknown or debated.

All model variants in the ensemble are deterministic. In the same way as the choice of a simple core structure for the models, we also opted for a simple translation of the network structure into a dynamic model. Although stochastic effects play a pivotal role in gene expression, the role of stochasticity in the other processes involved in diauxic growth, which involve a large number of molecules, have been less studied. Stochastic effects are especially important in (positive) feedback control loops and may in certain conditions lead to sub-populations growing on different carbon sources [30]. Also, the temporal behaviour of diauxic growth may be influenced by stochastic effects and, for example, affect the duration of the lag phase between glucose and a secondary carbon source [31–33]. Stochastic models resembling some of the variants in the model ensemble considered here have been published in the literature [31, 34]. However, given that the models most concerned by possible stochastic processes, kinetic models with regulation, are already among the best-scoring models, we do not believe that the use of stochastic extensions for these models would have affected the overall model ranking.

All model variants are capable

of reproducing a basic manifestation of CCR, namely diauxic growth on two alternative substrates. Fundamentally, this capacity resides in breaking the symmetry between the pathways for the two alternative substrates, by tuning kinetic parameters, stoichiometric coefficients, or costs/benefits assigned to the enzymes. By introducing a quantitative measure of diauxic growth *d*, the diauxic growth index, we investigated to which extent the models could be tuned so as to exhibit a perfect diauxic growth phenotype. The index is based solely on observations of the bio-reactor system, that is, the extracellular environment. In this way, the different mechanisms are assessed only by their output and not by intracellular structures or parameters, which allows an unbiased comparison of the large variety of model variants in the ensemble.

We observed that regulatory models, in general, show the highest values of *d*: three regulatory models rank under the top-five models, whereas constraint-based models rank between place 7 and 15, and resource allocation models between place 10 and 19, with the exception of model A2. This indicates that the dynamics of gene expression play a crucial role in CCR. Flux balance models in which the dynamics of gene expression are ignored lead to unsatisfactory behaviour (C3). Also, models taking into account competition for an energy-carrier metabolite necessary for transport (PTS or non-PTS variants) give a poor result: for example, the PTS model (A5), even after fine-tuning of the parameters, reaches a maximum value of *d*=0.59 only. Since a prerequisite for the choice of kinetic parameters was that growth should be possible on a single substrate, the choice of parameters is quite constrained and limits the possibility to obtain diauxic growth.

While it was possible to reproduce the standard experiment, consisting of diauxic growth of *E. coli* on minimal medium with glucose and lactose with almost all model variants, further data sets from [14] were used for model inference. A glucose pulse experiment and an experiment with a high initial concentration of LacZ were simulated using the model variants and a simple clustering procedure was carried out to group the predicted time-course profiles of lactose and LacZ and compare these with experimental data on a number of qualitative features. It turned out that the model variants based on well-known mechanisms (inducer exclusion and activation by a global transcription factor) show a very good performance and outperform other model variants. This does not rule out any causal effect on CCR by the factors underlying the latter models, but suggest that they complement rather than replace inducer exclusion and transcription factor activation.

In studies of diauxic growth, it is generally assumed that the substrate with the higher maximal growth rate *μ*_*max*_ is also the preferred substrate. This is the case for carbohydrates in *E. coli*. However, as a counter-example, it was reported for *Aromatoleum aromaticum* EbN1 that the organism prefers the substrate benzoate over succinate, whereas initial growth on benzoate leads to a slower growth rate than growth on succinate after the depletion of benzoate (Fig. 1A in [35]). We analysed our model ensemble to see if there are any model variants in which the preferred substrate could lead to a lower maximal growth rate. As expected, model variants with regulatory interactions could be quickly adjusted to represent this observation. Also, model variants with an objective function maximizing the growth rate obviously fail to reproduce this inverse diauxie. It appears, however, that model variant A2, based on the distribution of cellular resources for enzyme synthesis *via* the solution of an optimal control problem, prefers the substrate with the lower maximal growth rate if the upper bound for metabolite *M* is appropriately adjusted. Further analysis of the model ensemble could be carried out with other data sets, for example on the co-utilization of substrates in certain scenarios [9, 23].

In addition to clarifying the role played by different mechanisms in CCR, and function as a guide for experimental design, the collection of mathematical models presented here can also serve as an educational resource. The broad spectrum of available modelling techniques and tools for model analysis can be profitably applied, compared, and evaluated with the help of a simple and manageable - but relevant - example from systems biology.

## Additional file

Additional file 1 Additional file (Pdf format) includes the following sections: 1. Rescaled model equations, 2. Detailed description of model ensemble, 3. Results of cluster analysis, 4. Model fitting. (PDF 699 kb)

## Abbreviations

CCR: Carbon catabolite repression
FBA: Flux balance analysis
ODE: Ordinary differential equation
PTS: Phosphotransferase system
