# Supplementary material for: An ensemble of mathematical models showing diauxic growth behaviour
Source: BMC Syst Biol. 2018 Sep 21;12:82. doi: 10.1186/s12918-018-0604-8 (PMC6151013; doi:10.1186/s12918-018-0604-8)
Supplement: Supplementary file 1 — Additional file (Pdf format) includes the following sections: 1. Rescaled model equations, 2. Detailed description of model ensemble, 3. Results of cluster analysis, 4. Model fitting. (PDF 699 kb) [file 12918_2018_604_MOESM1_ESM.pdf]

Additional File – Supplementary Material

An ensemble of mathematical models showing diauxic growth behaviour

A. Kremling, Systems Biotechnology, Technische Universität München. Boltzmannstrasse 15, D - 85748 Garching, Germany
Mail: a.kremling@tum.de

J. Geiselmann, Laboratoire Interdisciplinaire de Physique, Université Grenoble Alpes, CNRS UMR5588. 140 avenue de la Physique, BP 87, 38402 Saint Martin d'Hères, France
Mail: johannes.geiselmann@univ-grenoble-alpes.fr

D. Ropers, Inria, Université Grenoble Alpes. 655 avenue de l'Europe, Montbonnot, 38334 Saint Ismier Cedex, France
Mail: delphine.ropers@inria.fr

H. de Jong, Inria, Université Grenoble Alpes. 655 avenue de l'Europe, Montbonnot, 38334 Saint Ismier Cedex, France
Mail: hidde.de-jong@inria.fr

A.1 Rescaled model equations

The following relations are used (lower-case letters denote dimensionless variables):

$$
\begin{aligned}
s_i &= \frac{S_i}{K_i}\,, \\
b &= \frac{B}{K_1\,Y_1}\,w_1\,, \\
e_i &= \frac{E_i}{Y_1}\,, \\
x_i &= \frac{X_i}{k_{s1}}\,Y_1\,k_{xi}\,, \\
m &= \frac{M}{k_{s1}}\,Y_1\,k_m\,, \\
b' &= B'\,Y_1\,, \\
\tau &= k_{s1}\,t\,,
\end{aligned}
\tag{A.1}
$$

and the following system of ODEs is obtained (the derivative is with respect to dimensionless time τ):

$$
\begin{aligned}
\dot{s}_1 &= -\frac{e_1\,s_1}{1\,+\,s_1}\,b\,, \qquad s_1(0) = s_{10}\,, \\
\dot{s}_2 &= -k_s\,K_s\,w_s\,\frac{e_2\,s_2}{1\,+\,s_2}\,b\,, \qquad s_2(0) = s_{20}\,, \\
\dot{b} &= \mu\,b\,, \qquad b(0) = b_0\,, \\
\dot{e}_1 &= k_{e1}\,f_1\,-\,\mu\,e_1\,, \qquad e_1(0) = e_{10}\,, \\
\dot{e}_2 &= k_{e2}\,f_2\,-\,\mu\,e_2\,, \qquad e_2(0) = e_{20} \\
\dot{x}_1 &= \frac{e_1\,s_1}{1\,+\,s_1}\,-\,x_1\,, \qquad x_1(0) = x_{10} \\
\dot{x}_2 &= k_s\,\frac{e_2\,s_2}{1\,+\,s_2}\,-\,x_2\,, \qquad x_2(0) = x_{20} \\
\dot{m} &= x_1\,+\,x_2\,-\,m\,, \qquad m(0) = m_0 \\
\dot{b}' &= m\,-\,\mu\,b'\,, \qquad b'(0) = b'_0\,,
\end{aligned}
\tag{A.2}
$$

with the following abbreviations:

$$k_s = \frac{k_{s2}}{k_{s1}}, \quad K_s = \frac{K_{s1}}{K_{s2}}, \quad w_s = \frac{w_2}{w_1},$$

$$k_{e1} = Y_1 \frac{k_1}{k_{s1}}, \quad k_{e2} = Y_1 \frac{k_2}{k_{s1}}, \quad Y = \frac{Y_2}{Y_1} \tag{A.3}$$

and the specific growth rate μ:

$$\mu = \frac{e_1 \, s_1}{1 + s_1} + k_s \, Y \, \frac{e_2 \, s_2}{1 + s_2}. \tag{A.4}$$

To find the maximal value for d (see Results), parameters k_s, K_s, w_s, k_{e1}, k_{e2}, and Y are set to 1 (with exception of the models from group 2). Only the model specific parameters were changed for the optimization. For models in group 2, parameters k_2, k_{e2}, and the parameter for induction K_i^* (see below) were chosen in such a way, that growth on the less preffered substrate is still possible.

For metabolites with a fast dynamics, such as X_1, X_2 and M, one obtains for the basic model by applying the quasi-steady-state assumption:

$$x_1 = \frac{e_1 \, s_1}{1 + s_1}, \quad x_2 = \frac{e_2 \, s_2}{1 + s_2}, \quad m = x_1 + k_s \, x_2 . \tag{A.5}$$

Equations (A.2) together with (A.5) represent a system of differential equations that can be solved when kinetic parameters are plugged in.

A.2 Detailed description of model ensemble

In this section we give a detailed description of the models in the ensemble, by specifying which equations change with respect to the (rescaled) model structure in Supplementary Material A.1. In order to break the symmetry between the two substrate pathways, so that the models are able to produce a diauxic growth pattern, only a single parameter, function, or condition was changed in every case. The simulation results are shown for each model, starting from initial conditions in which the substrate concentrations are equal. The parameter values used are those maximizing the diauxic growth index defined by Equation (13) in the main text.

Constraint-based models

Model C1: FBA

The stoichiometry of the basic constraint-based model structure, given by Equations (1), (2), (11), and (12) in the main text, does not allow to break the symmetry between the two pathways and produce diauxic growth. However, motivated by the observed growth behaviour of *E. coli* in a minimal medium with glucose and acetate, a slight modification of the structure of the reaction network is sufficient to achieve this (Figure A.1). In particular, we use the following model:

$$N'\ \underline{x} \ = \ \begin{bmatrix} h(S_1) & -1 & 0 & 0 & 0 \\ 0 & 0 & 1 & h(S_2) & -1 \\ 0 & 1 & -1 & 0 & 0 \end{bmatrix} \begin{pmatrix} E_1 \\ r_{d1} \\ r_{d2} \\ E_2 \\ r_b \end{pmatrix} \ = \ \underline{0}\,. \tag{A.6}$$

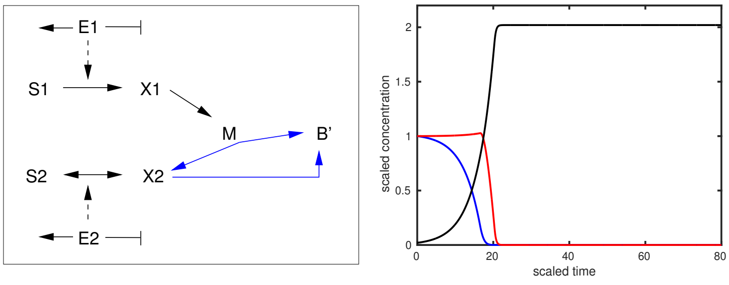

Figure A.1 Model **C1**. Left: reaction scheme. Right: simulation results for substrate S_1 (blue), substrate S_2 (red), and biomass B (black). The change in the structure of the reaction network is shown in blue.

Model C2: regulatory FBA

The regulatory constraints in regulatory FBA (rFBA) are modelled by logic rules relating environmental conditions to cellular responses, in particular the expression or activity of certain enzymes. For our model, we adapted a rule proposed in [1] for the growth of *E. coli* on glucose and lactose: `if` S_1 `is present then` E_2 `is not present`. The addition of this rule to the FBA model makes diauxic growth possible: the enzyme for S_2 uptake in the model is set to zero ($e_2 = 0$), if the glucose concentration is larger than a certain threshold value (Figure A.2).

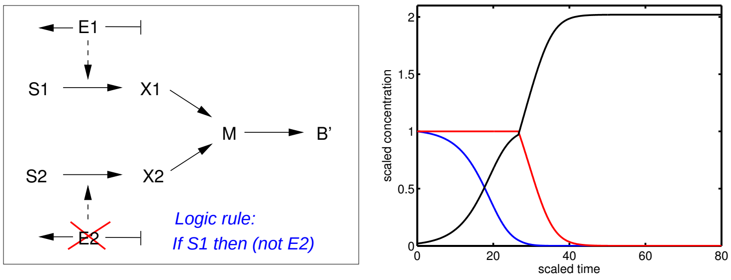

Figure A.2 Model **C2**. Left: reaction scheme. Right: simulation results for substrate S_1 (blue), substrate S_2 (red), and biomass B (black). The logic rule completing the structure of the reaction network is shown in blue.

Model C3: molecular crowding

The idea behind this modelling approach is that enzymes incorporated into the cell membrane occupy a certain fraction s of the cell volume. This results in an additional constraint on the enzyme concentrations [2], given by

$$\nu_1\ e_1\ +\ \nu_2\, e_2\ \leq\ s\ Vol\,, \tag{A.7}$$

where ν_1 and ν_2 are crowding coefficients and Vol is the maximal volume of the cell. Figure A.3 shows the reaction scheme and the simulation results obtained when adding Equation A.7 to the FBA model.

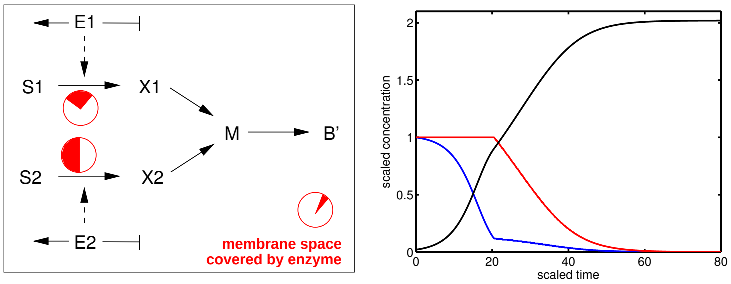

Figure A.3 Model **C3**. Left: reaction scheme. Right: simulation results for substrate S_1 (blue), substrate S_2 (red), and biomass B (black). The membrane space occupied by enzymes gives rise to an additional constraint.

Model C4: costs and benefits of enzymes

In this model variant, the costs to make enzymes and the benefits obtained (*e.g.*, increased extraction of energy from nutrients) are taken into account [3]. For example, the higher costs of a pathway (ATP necessary for enzyme synthesis) could be balanced by the higher benefits it yields (ATP generated). Costs and benefits are limited to a certain maximum amount Co:

$$\begin{bmatrix} \alpha_1 & \alpha_2 & \alpha_3 & \alpha_4 & \alpha_5 \end{bmatrix} \begin{pmatrix} e_1 \\ r_{d1} \\ e_2 \\ r_{d2} \\ r_b \end{pmatrix} \leq Co\,, \tag{A.8}$$

where α_i are cost coefficients. Rates of reactions for which the costs exceed the benefits (more ATP consumed than produced) have positive coefficients, whereas rates with higher benefits than costs (more ATP produced than consumed) have negative coefficients. In this example, we have $\alpha_1 > 0$, $\alpha_2 < 0$, $\alpha_3 > 0$, $\alpha_4 < 0$, and $\alpha_5 > 0$ (to reduce the number of freely selectable parameters for this model, we set $\alpha_1 = 1$, $\alpha_2 = -1$, $\alpha_4 = -1$, and $\alpha_5 = 0$. In this way, the model specific parameters that are changed in order to maximize d are α_3 and Co). Equations (11) in the main text and (A.8) together define the extended FBA problem that can be solved by linear programming. Figure A.4 shows the reaction scheme and the simulation results.

Kinetic models with growth dilution

The four models in this group are all variants of the basic kinetic model structure given by Equations (1)-(7). In addition, they all take into account (indirect) transcription regulation of the enzymes by their corresponding intracellular metabolites (inducers). This is modelled by changing f_i from its baseline value 1 to

$$f_i = \frac{x_i^n}{x_i^n + K_i^*}\,, \tag{A.9}$$

with parameters n and K_i^*.

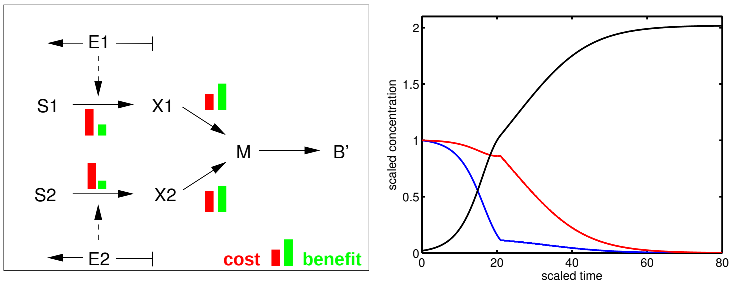

Figure A.4 Model **C4**. Left: reaction scheme. Right: simulation results for substrate S_1 (blue), substrate S_2 (red), and biomass B (black). The costs (red bars) and benefits (green bars) of reactions are shown.

Model N1: different maximum rates of enzyme synthesis

According to Narang and co-workers [4], different maximum synthesis rates k_{e1} and k_{e2} of the enzymes involved in the two pathways, while maintaining the same values for all other parameters, may lead to diauxic growth. Figure A.5 shows the reaction scheme and the simulation results obtained for such a model.

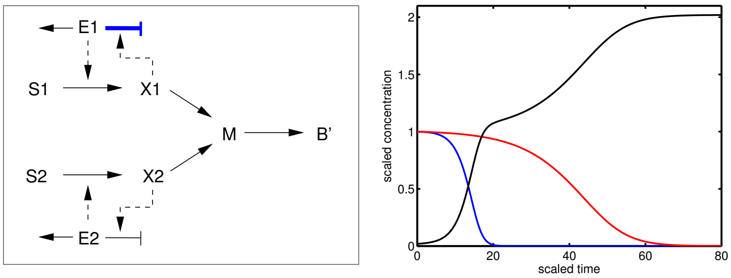

Figure A.5 Model **N1**. Left: reaction scheme. Right: simulation results for substrate S_1 (blue), substrate S_2 (red), and biomass B (black). The modified synthesis rate is marked in blue.

Model N2: different values for the initial enzyme concentrations

The system is simulated with the same set of parameters for the synthesis of the two enzymes, but the initial conditions $e_1(0) = e_1^0$ and $e_2(0) = e_2^0$ are different. Figure A.6 shows the reaction scheme and the simulation results.

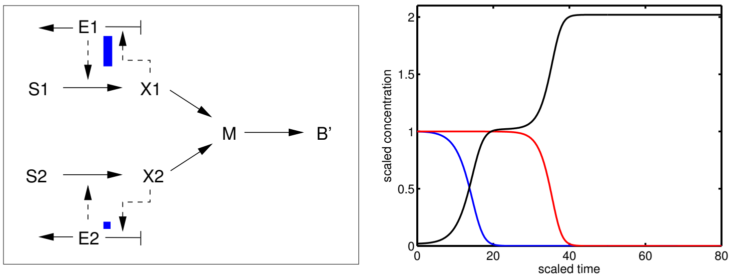

Figure A.6 Model **N2**. Left: reaction scheme. Right: simulation results for substrate S_1 (blue), substrate S_2 (red), and biomass B (black). The different initial conditions are marked in blue.

Model N3: different affinity for inducers

The inducers may have a different affinity for their respective repressor proteins, as reflected in the values of K_1^* and K_2^*, so that inducers differentially influence the synthesis of the two enzymes (Figure A.7).

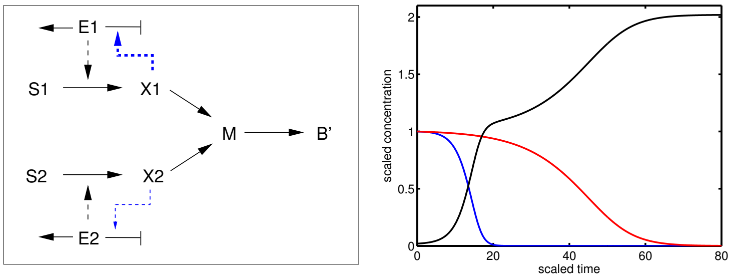

Figure A.7 Model **N3**. Left: reaction scheme. Right: simulation results for substrate S_1 (blue), substrate S_2 (red), and biomass B (black). The modified strength of the regulatory interaction is marked in blue.

Model N4: different maximum uptake rates for substrates

The turnover number of the transporters, and therefore the values of k_{s1} and k_{s2}, may be different and result in diauxic growth behaviour (Figure A.8).

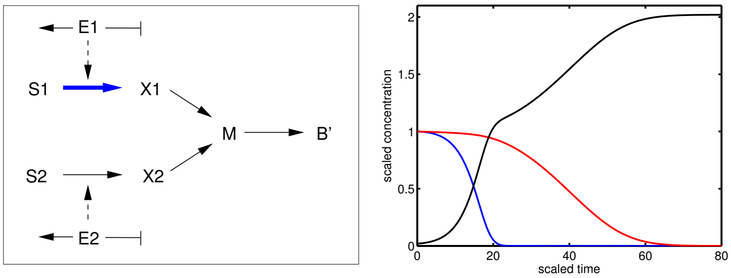

Figure A.8 Model **N4**. Left: reaction scheme. Right: simulation results for substrate S_1 (blue), substrate S_2 (red), and biomass B (black). The reaction with the modified (higher) maximum uptake rate is marked in blue.

Kinetic models taking into account metabolic and genetic regulation

Model R1: inducer exclusion

Inducer exclusion is the classical control scheme to prevent the influx of carbohydrates that may act as inducers. In the case of glucose uptake in *E. coli*, the activity of permease LacY is known to be inhibited by the non-phosphorylated form of one of the PTS components, the enzyme EIIA^{Glc}. In the simple model scheme used here, this is represented by an extension of the uptake rate r_{s2} to r^*_{s2}, involving the component X_1:

$$r^*_{s2} = r_{s2} \frac{K_I}{K_I + x_1} = \frac{e_1 \; s_1}{1 + s_1} \frac{K_I}{K_I + x_1} . \tag{A.10}$$

In this case X_1 has two functions in the simple model: inducer for the first enzyme and effector for the second one. Figure A.9 shows the reaction scheme and the simulation results.

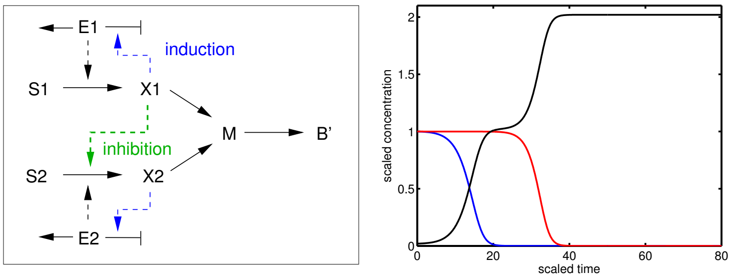

Figure A.9 Model **R1**. Left: reaction scheme. Right: simulation results for substrate S_1 (blue), substrate S_2 (red), and biomass B (black). The regulation of enzyme activity is shown in green.

Model R2: transcription factor activation

Transcription factor activation is modelled in the following way. In *E. coli* flux through central metabolism is sensed: according to the ratio of PEP and pyruvate the degree of phosphorylation of the PTS is altered. If the PEP/pyruvate ratio is high, protein Cya is activated by the phosphorylated form of protein EIIA^{Glc}. Due to the feed-forward structure in glycolysis (F1,6BP activates pyruvate kinase) a high

PEP/pyruvate ratio indicates low concentrations of central metabolites, whereas a low ratio corresponds to high concentrations. In the model, the rate of synthesis for the enzyme converting the second substrate is modified accordingly:

$$r_{e2}^{*} \;=\; k_{e2}\; f_2\; \frac{K_A}{K_A\; +\; m}\,. \tag{A.11}$$

In this way central metabolite M acts as an inhibitor for gene expression of the second enzyme. Figure A.10 shows the reaction scheme and the simulation results.

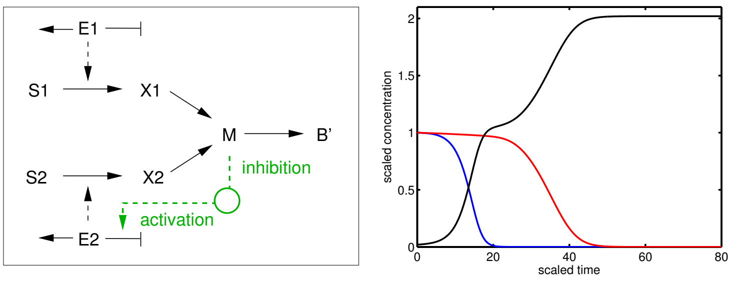

Figure A.10 Model **R2**. Left: reaction scheme. Right: simulation results for substrate S_1 (blue), substrate S_2 (red), and biomass B (black). The (indirect) inhibition of enzyme synthesis by a central metabolite is shown in green.

Models R3 and R4: central metabolite decreases/increases inducer concentration

Besides the classical and well-known schemes discussed here, further model variants can be imagined. As described in the main text, control schemes in central metabolic pathways might have an influence on metabolites which in turn can act as regulators of global transcription factors. The rate of enzyme synthesis strongly depends on the concentration of the inducer. Two model variants are considered: (i) In the model, the drain from metabolite X_2 may be a target of control. If metabolite M acts as an activator of this reaction, then the concentration of X_2 decreases with higher M, resulting in a lower rate of enzyme synthesis. (ii) Alternatively, M may act as an inhibitor of the reaction from X_1 to M. In this case, X_1 increases due to a higher concentration of M. Figures A.11 and A.12 show the reaction schemes for both model variants and the simulation results.

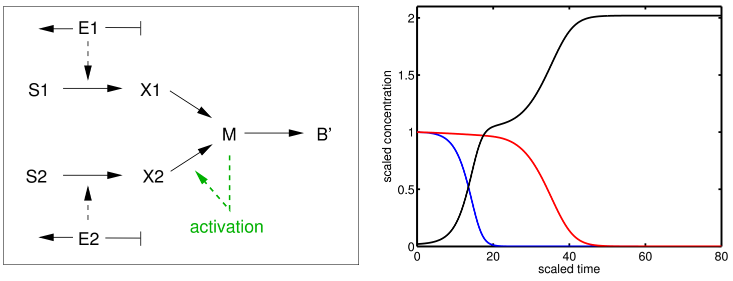

Figure A.11 Model **R3**. Left: reaction scheme. Right: simulation results for substrate S_1 (blue), substrate S_2 (red), and biomass B (black). The activation of the rate of the reaction consuming X_2 by a central metabolite is shown in green.

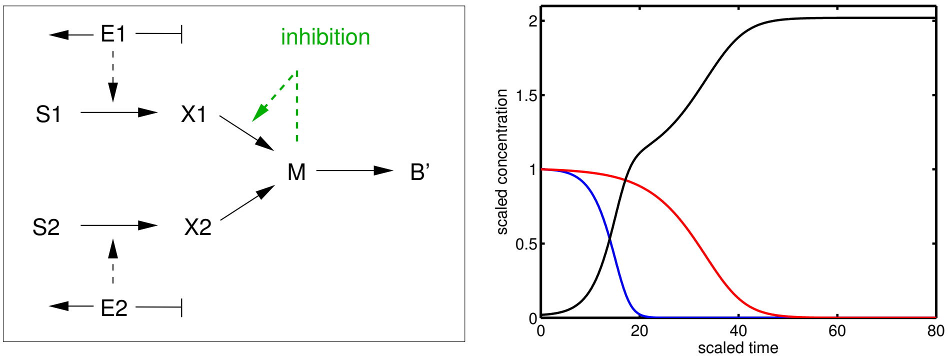

Figure A.12 Model **R4**. Left: reaction scheme. Right: simulation results for substrate S_1 (blue), substrate S_2 (red), and biomass B (black). The inhibition of the rate of the reaction consuming X_1 by a central metabolite is shown in green.

Resource allocation models

Models A1 and A2: distribution of macromolecular resources

In order to produce enzymes, resources in the form of amino acids as well as components of the transcriptional and translational apparatus are necessary. In the simple model, these different types of resources are all represented by the biomass compartment B'. Both model variants in this subsection are based on the availability of B', but they can be distinguished by the type of underlying optimization problem.

In model A1, a certain fraction of B' is available for transcription and translation of the enzymes (Figure A.13). As in the formulation of FBA, the growth rate is maximized. In this model, however, the growth rate is expressed as the sum of the substrate uptake rates:

$$r_{in} \;=\; r_{s1} + r_{s2}\,. \tag{A.12}$$

As explained in the Methods section of the main text, the rates are expressed in terms of the amount of enzyme ($\underline{e} = [e_1, e_2]^T$) and the corresponding optimization program reads:

$$\begin{aligned} \max \quad & r_{in} \;=\; [h(s_1)\;\; h(s_2)]\;\underline{e}\,, \\ \text{s.t.} \quad & [1\;1]\;\underline{e} \;\leq\; c\,b'\,, \end{aligned} \tag{A.13}$$

where c represents the fraction of b' available for the enzymes. This linear program can be solved at every time-point, for given values of s_1, s_2, and b', and the enzyme concentrations thus obtained are used as input for the dynamics of the remaining variables. The symmetry between the two substrate pathways is broken by changing the maximal uptake rate k_{s2} of the second substrate.

Model A2 is the dynamic variant of A1. In this case, the resources are consumed for the synthesis of the two enzymes (Figure A.14) and the objective is to maximize the biomass at t_{end}. In comparison with Equation (A.2), the functions f_1 and f_2 are changed to

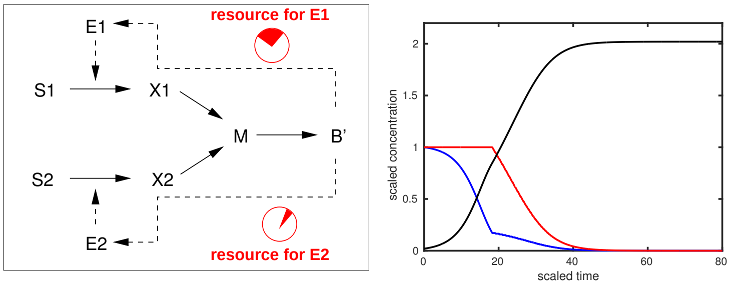

Figure A.13 Model **A1**. Left: reaction scheme. Right: simulation results for substrate S_1 (blue), substrate S_2 (red), and biomass B (black). The distribution of resources over the enzymes is shown by red circles.

$$f_1 \;=\; u\,b' \text{ and } f_2 \;=\; (u_{max} \;-\; u)\,b'\,. \tag{A.14}$$

This allows the distribution of a certain fraction ($u_{max} < 1$) of B' over the synthesis of the enzymes. The equation for the (scaled) biomass compartment b' takes into account the drain of resources to enzyme synthesis:

$$\dot{b}' \;=\; m \,-\, (u_{max} \,+\, \mu)\,b'\,, \qquad b'(0) = b'_0\,. \tag{A.15}$$

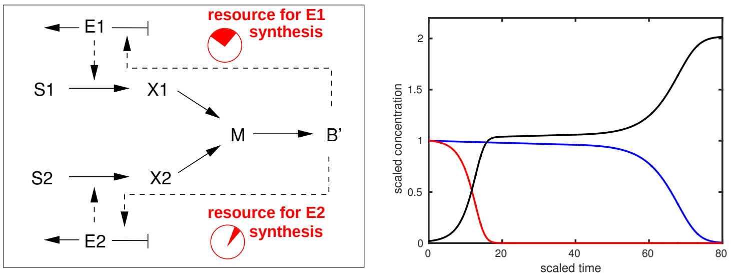

Figure A.14 Model **A2**. Left: reaction scheme. Right: simulation results for substrate S_1 (blue), substrate S_2 (red), and biomass B (black). The flow of resources to enzyme synthesis is shown by red circles.

The complete dynamic optimization program is then given by:

$$\begin{aligned} \max \qquad & b(t_{end}), \\ \text{s.t.} \quad \underline{\dot{x}} \;&=\; \underline{f}(\underline{x}, u)\,, \\ m \;&<\; m^*\,. \end{aligned} \tag{A.16}$$

with $\underline{x}$ are the state variables. The inequality constraint on m in the last equation guarantees that the concentration of this internal metabolite remains within physiological bounds. When simulating the system, it turns out that the dynamics are very sensitive to the value of this parameter m^*. When choosing low values for m^*, a diauxic growth pattern occurs without a change in the other parameters.

Model A3: cybernetic modelling approach

Nearly thirty years ago, Ramkrishna and co-workers proposed the cybernetic modelling framework [5, 6]. Like in model A2, the underlying idea is to allocate resources to enzyme synthesis depending on the carbon influx, but without solving an explicit optimization problem. Two so-called cybernetic variables are defined: u and v, where u_i represents the fractional allocation of resources to the synthesis of enzyme i given by $u_i = r_{si}/(r_{s1} + r_{s2})$ (matching law), and v_i represents enzyme activity control given by $v_i = r_{si}/\max(r_{s1}, r_{s2})$. To obtain diauxic growth, it is sufficient to consider only the control of enzyme synthesis (variables u_i). Figure A.15 shows the reaction scheme and the simulation results.

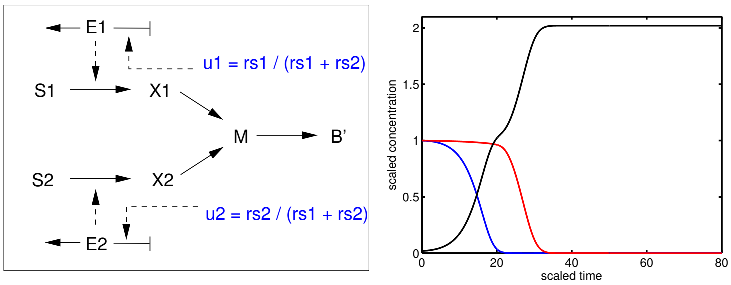

Figure A.15 Model **A3**. Left: reaction scheme. Right: simulation results for substrate S_1 (blue), substrate S_2 (red), and biomass B (black). The rate of enzyme synthesis is proportional to the fractional uptake rate of the corresponding substrates.

Models A4 and A5: competition for substrate transport resources

For the uptake of carbohydrates, energy in the form of ATP, a proton gradient or an internal metabolite is required (Figure A.16). Furthermore, some carbohydrates are taken up by a phosphotransferase system (PTS) in *E. coli* (Figure A.17). Therefore, two model variants are considered, one in which metabolite M is consumed as the energy source and one in which it used for the phosphorylation of transporter proteins. In order to compensate for the loss of M during substrate transport, in these models, the stoichiometry of the two reactions providing M from X_i is changed to 2 (one molecule of X_i provides two molecules of M). Notice that in these models the quasi-steady-state assumption for metabolite M is no longer valid and the differential equation instead of the algebraic equation for m is used. The equation for M read:

$$\dot{m} \;=\; 2\,(x_1 + x_2) - m - r_{s1} - r_{s2}\,, \qquad m(0) = m_0\,. \tag{A.17}$$

In model A4, where metabolite M is used as the energy source, the uptake rates for the substrates read:

$$\begin{aligned} r_{s1} &= e_1 \,\frac{s_1}{1 + s_1}\,\frac{m}{K_{M1} + m}\,, \\ r_{s2} &= k^*\, e_2\,\frac{s_2}{1 + S_2}\,\frac{m}{K_{M2} + m}\,. \end{aligned} \tag{A.18}$$

The steady-state values for x_1 and x_2 change accordingly. In order to break the symmetry between the two pathways, we change parameter k^* to a value lower than 1.

In model A5, the carbohydrates are taken up by a PTS, a group translocation system generally involving a number of proteins in either phosphorylated or unphosphorylated form. In order to mimic the behaviour

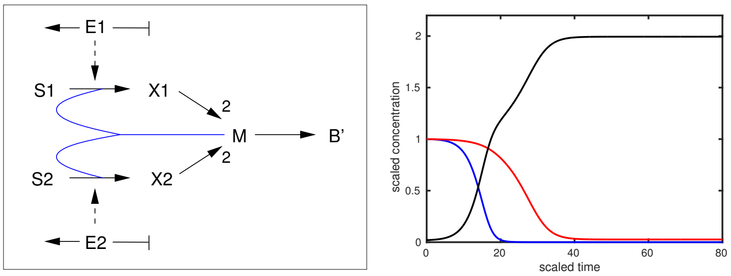

Figure A.16 Model **A4**. Left: reaction scheme. Right: simulation results for substrate S_1 (blue), substrate S_2 (red), and biomass B (black). M is used as as an energy source for the transport systems.

of a PTS with the simple model structure given here, we consider a phosphorylated form e_p and an unphosphorylated form e of the enzymes. The kinetic expressions for the uptake rates read:

$$
\begin{aligned}
r_{s1} &= e_{p1}\,\frac{s_1}{1\,+\,s_1}\,,\\
r_{s2} &= e_{p2}\,\frac{s_2}{1\,+\,S_2}\,,
\end{aligned}
\qquad (A.19)
$$

and the kinetic expressions for the regeneration of the phosphorylated forms read:

$$
\begin{aligned}
r_{m1} &= k_{p1}\,e_1\,m\,-\,k_{p_{m1}}\,e_{p1}\,,\\
r_{m2} &= k_{p2}\,e_2\,m\,-\,k_{p_{m2}}\,e_{p2}\,,
\end{aligned}
\qquad (A.20)
$$

For the unphosphorylated enzymes e_1 and e_2, only weak induction is taken into account. A difference in value of parameters k_{pi} allows to break the symmetry between the two substrate pathways. Induction and dilution are taken into account in the equations for the unphosphorylated proteins. The modified equations for the system read:

$$
\begin{aligned}
\dot{e}_1 &= k_{e1}\,f_1\,+\,r_{s1}\,-\,r_{m1}\,-\,\mu\,e_1\,, & e_1(0) &= e_{10}\,,\\
\dot{e}_2 &= k_{e2}\,f_2\,+\,r_{s2}\,-\,r_{m2}\,-\,\mu\,e_2\,, & e_2(0) &= e_{20}\\
\dot{m} &= 2\,(x_1\,+\,x_2)\,-\,m\,-\,r_{m1}\,-\,r_{m2}\,, & m(0) &= m_0\\
\dot{e}_{p1} &= r_{m1}\,-\,r_{s1}\,, & e_{p1}(0) &= e_{p10}\\
\dot{e}_{p2} &= r_{m2}\,-\,r_{s2}\,, & e_{p2}(0) &= e_{p20}
\end{aligned}
\qquad (A.21)
$$

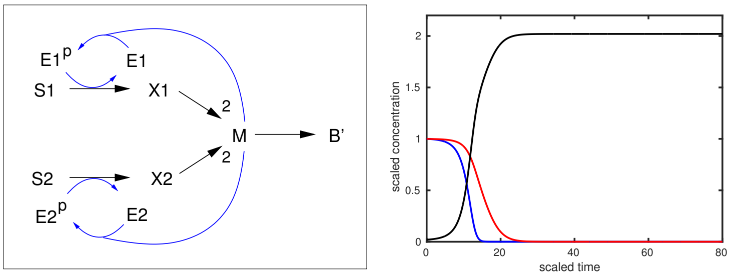

Figure A.17 Model **A5**. Left: reaction scheme. Right: simulation results for substrate S_1 (blue), substrate S_2 (red), and biomass B (black). Both PTS transport systems need M to take up the substrates.

Model A6: maximizing time

As discussed in [7], a possible cellular strategy is to maximize survival, which can be formulated as follows in the context of our modelling framework:

$$\begin{aligned} \max \quad & t_{end}\,, \\ \text{s.t.} \quad \dot{\underline{x}} &= \underline{f}(\underline{x}, u)\,, \\ b' &> b^*\,, \end{aligned} \tag{A.22}$$

where $\underline{x}$ represents the state variables in the model and where the last equation is a constraint to have the main biomass compartment larger than a threshold value. To solve the optimization problem, the Matlab package DOTcvp was used.

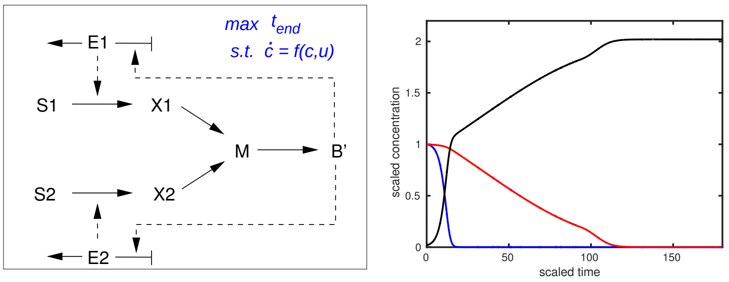

Figure A.18 Model **A6**. Left: reaction scheme. Right: simulation results for substrate S_1 (blue), substrate S_2 (red), and biomass B (black). The lifetime t_{end} is maximized.

A.3 Results of cluster analysis

Figure A.19 shows the dendrograms for the experiment with a high initial enzyme concentration and Figure A.20 shows the dendrograms for the pulse experiment. Moreover, Figure A.21 shows the time-courses of the mean of each cluster as well as of the individual models (in grey). Table A.1 summarizes how the models are divided between the clusters in the case of the experiment with a high initial enzyme concentration, while Table A.2 provides the same information for the pulse experiment.

We describe the procedure in more detail for the pulse experiment: the system is simulated over a short time period in the presence of lactose (substrate 2) and then the initial condition for glucose (substrate 1) is changed. The predicted time-courses of the substrate lactose and the enzyme for lactose uptake are stored in matrices for all model variants. In order to perform hierarchical clustering, the Matlab command "linkage" with options "ward" and "euclidean" is used. Finally the dendrogram is plotted.

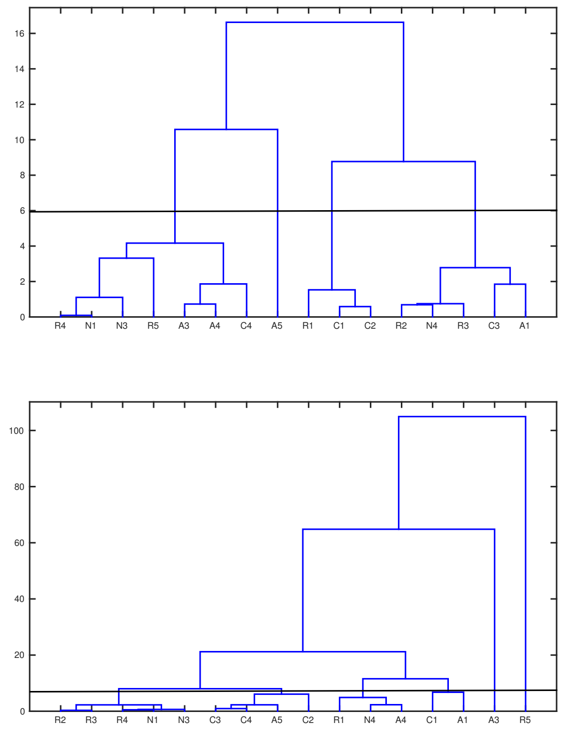

Figure A.19 Dendrograms for the experiment with a high initial enzyme concentration (top: substrate, bottom: enzyme).

We find a qualitative agreement with the experimental data for the following clusters:

- Experiment with a high initial enzyme concentration:
  - Substrate: Cluster 1
  - Enzyme: Clusters 1, 3, 5, and 6
- Pulse experiment:
  - Substrate: Clusters 2 and 3
  - Enzyme: Clusters 4, 5, and 6

As discussed in the main text, taking the intersection of the clusters consistent with the experimental data confirms that model variant R1 (inducer exclusion) is the main mechanism for glucose-lactose diauxic growth behaviour.

Table A.1 Models contained in each cluster (identified by its number) for the experiment with a high initial enzyme concentration.

Substrate		Enzyme	
Model	Cluster	Model	Cluster
R1	1	R2	1
C1	1	R3	1
C2	1	R4	1
R2	2	N1	1
R3	2	N3	1
N4	2	C3	2
C3	2	C2	2
A1	2	C4	2
A6	3	A6	2
R4	4	R1	3
N1	4	N4	3
N3	4	A5	3
A3	4	C1	4
C4	4	A1	4
A5	4	A3	5
R5	4	R5	6

Table A.2 Models contained in each cluster (identified by its number) for the pulse experiment.

Substrate		Enzyme	
Model	Cluster	Model	Cluster
R3	1	C3	1
N4	1	C4	1
A3	1	C2	2
A5	1	A1	2
R4	2	A6	2
N1	2	C1	3
C3	2	R1	4
C4	2	R2	4
R5	2	R3	4
R1	3	R4	4
R2	3	N1	4
C1	3	N3	4
C2	3	N4	4
A1	3	A5	4
A6	3	A3	5
N3	4	R5	6

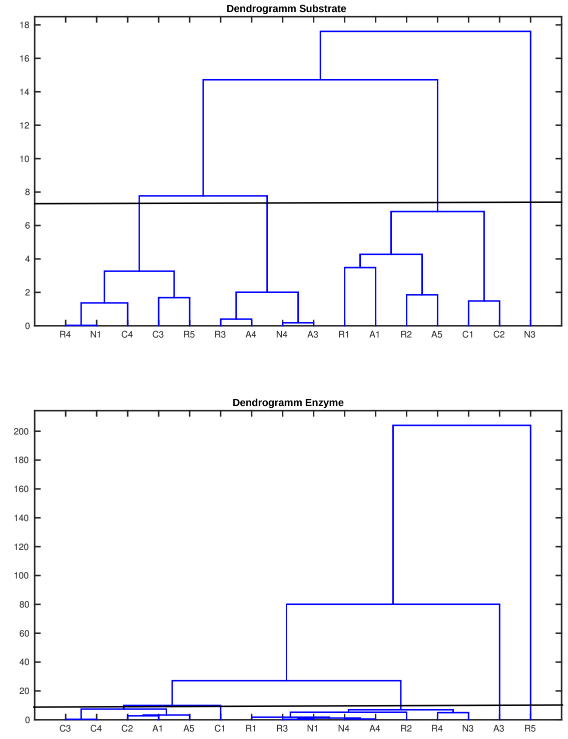

Figure A.20 Dendrograms for the pulse experiment (top: substrate, bottom: enzyme).

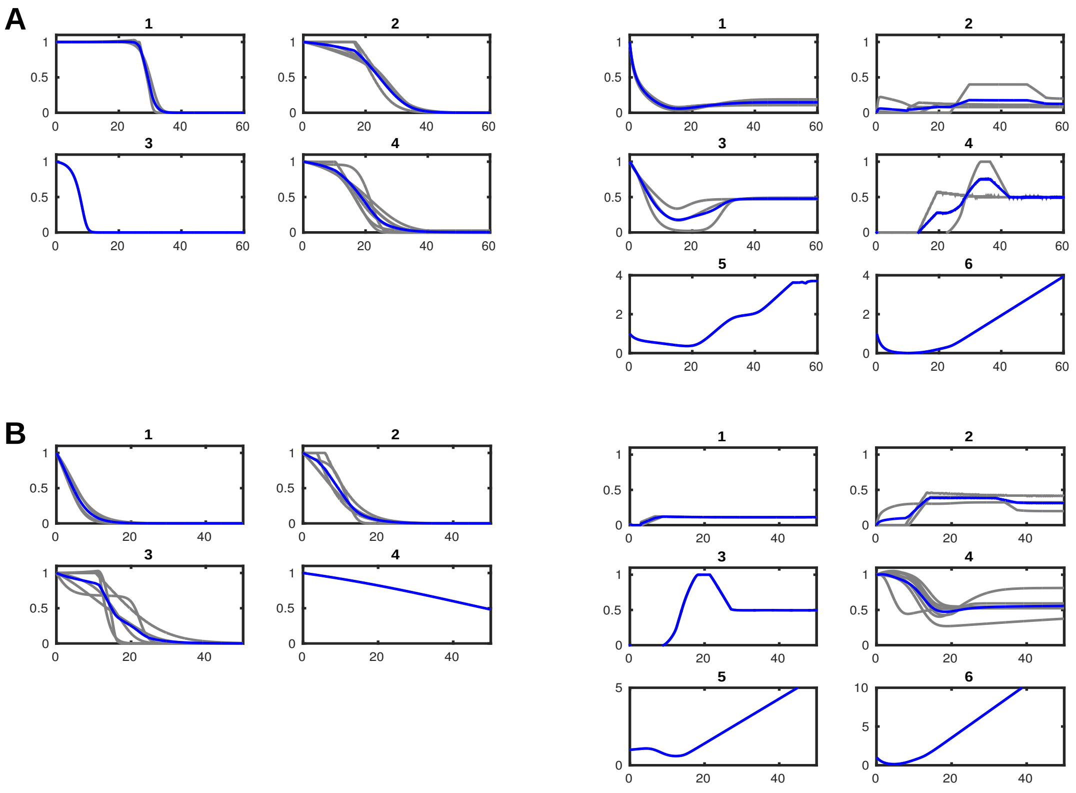

Figure A.21 A: Substrate (left) and enzyme (right) time-courses for the experiment with a high initial enzyme concentration. B: Idem for the pulse experiment. Each numbered plot corresponds to a cluser used in Tables A.1 and A.2.

A.4 Model fitting

The best set of parameters to describe the experimental data was obtained with a genetic algorithm with Matlab as describe in [8]. Parameters used are given in the follwing:

```
populationSize = 40;
numberOfGenes =  40;
crossoverProbability = 0.8;
mutationProbability = 0.025;
tournamentSelectionParameter = 0.75;
numberOfGenerations = 30;
```

The range for each paramter was selected independently.

Figure A.22 shows the fit of the models in the ensemble with the experimental data of the glucose-lactose diauxie from [9] and a statistical evaluation of the fit (χ^2-test). For glucose, 5 data points and for lactose and biomass 12 data points were used for model fitting. The measured error was estimated as 10% for glucose and 15% for lactose and biomass.

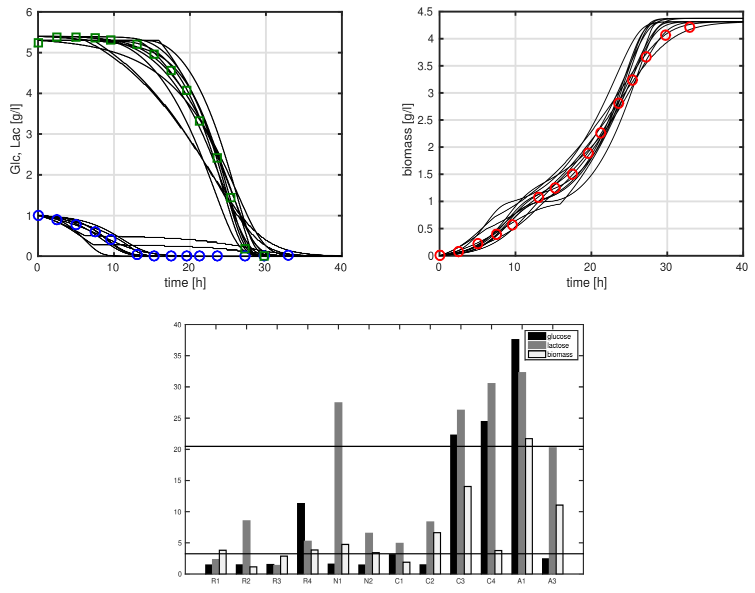

Figure A.22 Upper plots: comparision of the predicted time-courses for all fitted models (solid curves) with the experimentally-measured time-courses of glucose, lactose, and biomass [9]. Lower plot: values of the objective function (bars) and threshold of χ^2 statistics (solid lines).

Author details

References

1. Covert, M.W., Xiao, N., Chen, T.J., Karr, J.R.: Integrating metabolic, transcriptional regulatory and signal transduction models in *Escherichia coli*. Bioinformatics **24**(18), 2044–50 (2008)
2. Beg, Q.K., Vazquez, A., Ernst, J., de Menezes, M.A., Bar-Joseph, Z., Barabasi, A.L., Oltvai, Z.N.: Intracellular crowding defines the mode and sequence of substrate uptake by *Escherichia coli* and constrains its metabolic activity. Proc. Natl. Acad. Sci. U.S.A. **104**(31), 12663–8 (2007)
3. Wessely, F., Bartl, M., Guthke, R., Li, P., Schuster, S., Kaleta, C.: Optimal regulatory strategies for metabolic pathways in *Escherichia coli* depending on protein costs. Mol. Syst. Biol. **7**, 515 (2011)
4. Narang, A., Pilyugin, S.S.: Bacterial gene regulation in diauxic and non-diauxic growth. J. Theor. Biol. **244**(2), 326–48 (2007)
5. Ramakrishna, R., Ramkrishna, D., Konopka, A.E.: Cybernetic modeling of growth in mixed, substitutable substrate environments: Preferential and simultaneous utilization. Biotechnol. Bioeng. **52**(1), 141–51 (1996)
6. Ramkrishna, D., Song, H.-S..: Dynamic models of metabolism: review of the cybernetic approach. AIChE J. **58**(4), 1160–6 (2012)

7. de Hijas-Liste, G.M., Klipp, E., Balsa-Canto, E., Banga, J.: Global dynamic optimization approach to predict activation in metabolic pathways. BMC Syst. Biol. **8**(1), 1 (2014)
8. Wahde, M., Sandberg, D., Benderius, O.: An Elementary Introduction to Matlab Programming for Stochastic Optimization. (2015). http://www.documbase.com/LAB-1%3A-MATLAB-Introduction-to-Programming.pdf
9. Bettenbrock, K., Fischer, S., Kremling, A., Jahreis, K., Sauter, T., Gilles, E.D.: A quantitative approach to catabolite repression in *Escherichia coli*. J. Biol.Chem. **281**, 2578–84 (2006)
